# Supplementary material for: A framework for deriving analytic steady states of biochemical reaction networks
Source: PLoS Comput Biol. 2023 Apr 13;19(4):e1011039. doi: 10.1371/journal.pcbi.1011039 (PMC10129002; doi:10.1371/journal.pcbi.1011039)
Supplement: S1 Text — (PDF) [file pcbi.1011039.s001.pdf]

S1 Text for A framework for deriving analytic steady states of biochemical reaction networks

Bryan S. Hernandez
Biomedical Mathematics Group, Pioneer Research Center for Mathematical and Computational Sciences, Institute for Basic Science, Daejeon 34126, Republic of Korea[*]

Patrick Vincent N. Lubenia
Systems and Computational Biology Research Unit, Center for Natural Sciences and Environmental Research, 2401 Taft Avenue, Manila, 0922, Metro Manila, Philippines

Matthew D. Johnston
Department of Mathematics and Computer Science, Lawrence Technological University, Southfield, Michigan 48075, United States of America

Jae Kyoung Kim[†]
Department of Mathematical Sciences, KAIST, Daejeon 34141, Republic of Korea[‡]

(Dated: March 24, 2023)

SUPPLEMENTARY METHODS

Independent decompositions and positive steady states

Let $\mathcal{N}$ be a CRN with reaction set $\mathcal{R}$. We associate a kinetics $\mathcal{K}$ to the network $\mathcal{N}$. Let $x = x(t) \in \mathbb{R}^m_{\geq 0}$ be the vector of the concentrations of the m species in the network according to the associated kinetics at time t. Then, the ODEs for the chemical reaction system (i.e., CRN $\mathcal{N}$ with the kinetics $\mathcal{K}$) can be written as

$$f(x) = \sum_{y \to y' \in \mathcal{R}} \mathcal{K}_{y \to y'}(x)(y' - y)$$

where $\mathcal{K}_{y \to y'}$ is the kinetics term for the reaction $y \to y' \in \mathcal{R}$.

Since $f(x)$ is the sum of scalar multiples of the reaction vectors, then $f(x) \in S$ where $S = \text{span}\{y' - y | y \to y' \in \mathcal{R}\}$, i.e., the span of the reaction vectors called the *stoichiometric subspace* of the CRN.

Suppose we decompose the CRN $\mathcal{N}$ into α subnetworks; then we can regroup the terms of the ODEs in the following manner:

$$\begin{aligned} f(x) &= \sum_{y \to y' \in \mathcal{R}} \mathcal{K}_{y \to y'}(x)(y' - y) \\ &= \sum_{y \to y' \in \mathcal{R}_1} \mathcal{K}_{y \to y'}(x)(y' - y) + \cdots + \sum_{y \to y' \in \mathcal{R}_\alpha} \mathcal{K}_{y \to y'}(x)(y' - y) \\ &= f_1(x) + \cdots + f_\alpha(x) \end{aligned}$$

where $\mathcal{R}_1, \mathcal{R}_2, \ldots, \mathcal{R}_\alpha$ are the reaction sets of subnetworks $\mathcal{N}_1, \mathcal{N}_2, \ldots, \mathcal{N}_\alpha$, respectively.

Now, suppose that c is a positive steady state of each subsystem $(\mathcal{N}_i, \mathcal{K}_i)$; then $f_i(c) = 0$ for each i. So, $f(c) = 0$ and c is a positive steady state of the whole system $(\mathcal{N}, \mathcal{K})$. Thus,

$$E_1 \cap E_2 \cap \cdots \cap E_\alpha \subseteq E$$

where E is the set of positive steady states of the whole system and E_i is the set of positive steady states of the ith subsystem.

---

[*] Also at Institute of Mathematics, University of the Philippines Diliman, Quezon City 1101, Philippines
[†] jaekkim@kaist.ac.kr
[‡] Also at Biomedical Mathematics Group, Pioneer Research Center for Mathematical and Computational Sciences, Institute for Basic Science, Daejeon 34126, Republic of Korea

Conversely, suppose c is a positive steady state of the whole system; then $f(c) = f_1(c) + \cdots + f_\alpha(c) = 0 = 0 + \cdots + 0$ where

$$f_i(c) = \sum_{y \to y' \in \mathcal{R}_i} \mathcal{K}_{y \to y'}(c)(y' - y) \subseteq S_i$$

and $S_i = \text{span}\{y' - y | y \to y' \in \mathcal{R}_i\}$. If the induced subnetworks are independent, then the stoichiometric subspace of the whole network is the direct sum of the the stoichiometric subspaces of its subnetworks, i.e., $S = S_1 \oplus \cdots \oplus S_\alpha$. Thus, $f_i(c) = 0$ for each i, i.e., c is a positive steady state of $(\mathcal{N}_i, \mathcal{K}_i)$ for each i. Therefore,

$$E \subseteq E_1 \cap E_2 \cap \cdots \cap E_\alpha.$$

The argument above leads to the following decomposition theorem by Martin Feinberg [1, 2]:

Theorem 1. *Let $(\mathcal{N}, \mathcal{K})$ be a reaction system decomposed into subsystems $(\mathcal{N}_1, \mathcal{K}_1)$, $(\mathcal{N}_2, \mathcal{K}_2), \ldots, (\mathcal{N}_\alpha, \mathcal{K}_\alpha)$ induced by the decomposition of the underlying network $\mathcal{N}$ into $\{\mathcal{N}_1, \mathcal{N}_2, ..., \mathcal{N}_\alpha\}$ and $\mathcal{K}_i$ be the restriction of $\mathcal{K}$ to reactions in $\mathcal{R}_i$. Then*

$$E_1 \cap E_2 \cap \cdots \cap E_\alpha \subseteq E.$$

If the network decomposition is independent, then equality holds, i.e.,

$$E_1 \cap E_2 \cap \cdots \cap E_\alpha = E.$$

To utilize the latter part of Theorem 1, we must identify an independent decomposition of a given CRN. This was addressed in a paper by B. Hernandez and R. de la Cruz [3], where they provided a necessary and sufficient condition on the existence of independent decompositions of CRNs. Importantly, they established an efficient method of finding such decompositions. Recently, Hernandez et al. [4] showed that the independent decomposition obtained from this method is the finest, which means that one can no longer further decompose the decomposed network to get another independent decomposition with a higher number of subnetworks. Furthermore, such finest decomposition can be easily obtained via programs in Octave and MATLAB that were created by P. Lubenia [5].

Aside from utilizing Theorem 1 via the algorithm developed previously, one should also consider whether the steady states of the subnetworks can always be merged to get the steady state of the whole network regardless of the choice of rate constants. We address this problem with the following result that provides a sufficient condition for the existence of a positive steady state of the whole network for all rate constants by merging the positive steady states of its independent subnetworks. The proof was patterned on a work by B. Boros [6], which considers only a specific type of independent decomposition of a CRN into its linkage classes. Our theorem, however, considers any type of independent decomposition.

Theorem 2. *Let a CRN $\mathcal{N}$ under mass-action kinetics be decomposed into d independent subnetworks such that $\delta = \delta_1 + \cdots + \delta_d$ and $\widehat{T}$-independent (i.e., the augmented matrix of source nodes of $\mathcal{N}$ is the direct sum of the augmented matrices of source nodes of its subnetworks). If $E_j \neq \varnothing$ for $j = 1, \ldots, d$, then $E = \bigcap_{j=1}^{d} E_j \neq \varnothing$.*

Proof. Suppose $E_j \neq \varnothing$ for all j. By the alternative definition of a steady state (see page 11 of [6]),

$$x \in E_j \iff \theta_j(x) = v_j \text{ and } Y_j \cdot I_j \cdot v_j = 0 \text{ for some } v_j \in \mathbb{R}^{n_j}_{>0}$$

where v_j is a positive vector where the number of components is the number of nodes (n_j) in subnetwork j, θ_j is the matrix of monomials associated with the nodes, Y_j is the matrix of nodes and I_j is the Laplacian matrix of labeled reactions by the rate constants in subnetwork j (see page 10 of [6]). We emphasize that the existence of the positive steady state does not depend on the rate constants because the positive vector v_j does not depend on the rate constants either.

Since $\log(\theta_j(x)) = (T_j)^\top \log(x)$ and $\log : \mathbb{R}^m_{>0} \to \mathbb{R}^m$ is bijective,

$$x \in E_j \iff \log(v_j) \in \text{Im}(T_j)^\top \text{ and } Y_j \cdot I_j \cdot v_j = 0 \text{ for some } v_j \in \mathbb{R}^{n_j}_{>0}.$$

Let $\mathbf{1}_j = (1, 1, \ldots, 1) \in \mathbb{R}^{n_j}_{>0}$. Since $\text{Im}(\widehat{T}_j)^\top = \text{Im}\left[(T_j)^\top, \mathbf{1}_j\right]$ and $\log(\gamma_j v_j) = \log(v_j) + \log(\gamma_j)\mathbf{1}_j$ for $\gamma_j \in \mathbb{R}_{>0}$, $\log(v_j) \in \text{Im}(T_j)^\top \iff \log(v_j) \in \text{Im}(\widehat{T}_j)^\top$. Therefore,

$$x \in E_j \iff \log(v_j) \in \text{Im}(\widehat{T}_j)^\top \text{ and } Y_j \cdot I_j \cdot v_j = 0 \text{ for some } v_j \in \mathbb{R}^{n_j}_{>0}.$$

Then, for each j, fix v_j such that $\log(v_j) \in \operatorname{Im}(\widehat{T}_j)^\top$ and $Y_j \cdot I_j \cdot v_j = 0$. By assumption, the decomposition is $\widehat{T}$-independent. Thus, by Corollary B.2 of [6], we have

$$\bigcap_{i=1}^{d} \left\{ z \in \mathbb{R}_{>0}^{m+1} | \left(\widehat{T}_j\right)^\top \cdot z = \log(v_j) \right\} \neq \varnothing.$$

Hence,

$$\begin{bmatrix} \log(v_1) \\ \vdots \\ \log(v_d) \end{bmatrix} = \begin{bmatrix} \left(\widehat{T}_1\right)^\top \\ \vdots \\ \left(\widehat{T}_d\right)^\top \end{bmatrix} \begin{bmatrix} u \\ w \end{bmatrix}$$

for some $u \in \mathbb{R}^m$ and $w \in \mathbb{R}^d$. Let $x \in \mathbb{R}^n_{>0}$ and $\gamma_j \in \mathbb{R}_{>0}$ such that $\log(x) = u$ and $-\log(\gamma_j) = w_j$ for $j = 1, \ldots, d$. Hence,

$$\begin{bmatrix} \log(v_1) \\ \vdots \\ \log(v_d) \end{bmatrix} = \begin{bmatrix} \left(\widehat{T}_1\right)^\top \\ \vdots \\ \left(\widehat{T}_d\right)^\top \end{bmatrix} \begin{bmatrix} \log(x)^\top \\ -\log(\gamma_1) \\ \vdots \\ -\log(\gamma_d) \end{bmatrix}.$$

Thus, for all j and for all $y \in \mathcal{C}_j$, we have $\gamma_j v_j^y = \prod_{s=1}^{m} x_s^{T_{sy}} = \theta_j^y(x)$, which yields $\gamma_j v_j = \theta_j(x)$. Then,

$$\begin{aligned} Y \cdot I \cdot \theta(x) &= \sum_{j=1}^{d} Y_j \cdot I_j \cdot \theta_j(x) \\ &= \sum_{j=1}^{d} \gamma_j \cdot Y_j \cdot I_j \cdot v_j \\ &= \sum_{j=1}^{d} \gamma_j \cdot 0 \\ &= 0, \end{aligned}$$

which implies that $x \in E$, and hence $E \neq \varnothing$. Since the decomposition is independent and by Theorem 1, $\bigcap_{j=1}^{d} E_j = E$.

Therefore, $E = \bigcap_{j=1}^{d} E_j \neq \varnothing$. □

Example 1. Consider the toy example in Fig 1 in the main text, which is shown again in Fig A. The CRN $\mathcal{N}$ is decomposed into two independent subnetworks $\mathcal{N}_1$ and $\mathcal{N}_2$. We can easily show that the sum of the deficiencies of these two subnetworks ($\mathcal{N}_1$ and $\mathcal{N}_2$) sum up to the deficiency of the the whole network ($\mathcal{N}$). In the figure, we compute the the matrix of source nodes of the network $\mathcal{N}$ (denoted by $\widehat{T}$) and its subnetworks $\mathcal{N}_1$ and $\mathcal{N}_2$ (denoted by $\widehat{T}_1$ and $\widehat{T}_2$, respectively). It is also easy to compute that the rank of the matrix of source nodes of the whole network is equal to the sum of the ranks of the matrices of source nodes of its subnetworks, i.e., rank $\widehat{T} = 6 = 2+4 =$ rank $\widehat{T}_1+$rank $\widehat{T}_2$. Hence by Theorem 2, the intersection of the set of positive steady states of the whole network is nonempty for all rate constants. This is reason why we were able to successfully merge the positive steady states of the two subnetworks and obtain the positive steady state of the whole network for all positive rate constants as shown in Fig 1 in the main text.

In contrast, the following CRN has a positive steady state only for limited values of rate constants.

Example 2. Consider the CRN in Fig B (upper left). We decompose the network $\mathcal{N}$ into two independent subnetworks $\mathcal{N}_1$ and $\mathcal{N}_2$ (upper right). Then, we can easily compute the steady state of each subnetwork and obtain $\left(\tau_1, \frac{k_1}{k_2}\tau_1\right)$ for $\mathcal{N}_1$ and $\left(\tau_2, \sqrt{\frac{k_3}{k_4}}\tau_2\right)$ for $\mathcal{N}_2$. To get the steady state of $\mathcal{N}$, the steady states of $\mathcal{N}_1$ and $\mathcal{N}_2$ must coincide. We can only merge these two steady states successfully whenever $\frac{k_1}{k_2} = \sqrt{\frac{k_3}{k_4}}$. Hence, $\mathcal{N}$ has a positive steady state only for limited rate constants.

A CRISPRi toggle switch model

The reactions and the species in the CRISPRi toggle switch model are provided in Fig 3 in the main text and Table A, respectively. As discussed in the main text, the steady state concentration of species S (single guide RNA 1) is given by

$$s = \frac{Q + \sqrt{Q^2 + 4 \cdot \dfrac{k_1 k_3 k_7 k_{12}(g_0 + r_0)}{k_4 k_8 k_{11} k_{13}}}}{2 \cdot \dfrac{k_3 k_7 k_{12}}{k_4 k_8 k_{11}}}$$

where

$$Q = -\left(1 + \frac{k_2 k_5 k_9 k_{12}(h_0 + p_0)}{k_6 k_{10} k_{11} k_{14}}\right) + \frac{k_1 k_3 k_7 k_{12}(g_0 + r_0)}{k_4 k_8 k_{11} k_{13}}.$$

We already established the monotonicity of s over k_1, k_2, k_5, k_6, k_9, k_{10}, k_{13} and k_{14} (main text). Here, we determine its monotonicity over k_3, and a similar approach can be used for the remaining positive rate constants. Manipulating this steady state equation for s, we get

$$\begin{aligned} s =& \frac{Q}{2 \cdot \dfrac{k_7 k_{12}}{k_4 k_8 k_{11}}(k_3)} + \frac{\sqrt{Q^2 + 4 \cdot \dfrac{k_1 k_7 k_{12}(g_0 + r_0)}{k_4 k_8 k_{11} k_{13}}(k_3)}}{\sqrt{\left(2 \cdot \dfrac{k_7 k_{12}}{k_4 k_8 k_{11}}(k_3)\right)^2}} \\ =& \frac{Q}{2 \cdot \dfrac{k_7 k_{12}}{k_4 k_8 k_{11}}(k_3)} + \sqrt{\left(\frac{Q}{2 \cdot \dfrac{k_7 k_{12}}{k_4 k_8 k_{11}}(k_3)}\right)^2 + \frac{k_1 k_4 k_8 k_{11}(g_0 + r_0)}{k_7 k_{12} k_{13}(k_3)}}. \end{aligned}$$

Meanwhile, by substituting the value of Q to the first term in the expression above, we have

$$\begin{aligned} \frac{Q}{2 \cdot \dfrac{k_7 k_{12}}{k_4 k_8 k_{11}}(k_3)} =& \frac{-\left(1 + \dfrac{k_2 k_5 k_9 k_{12}(h_0 + p_0)}{k_6 k_{10} k_{11} k_{14}}\right) + \dfrac{k_1 k_7 k_{12}(g_0 + r_0)}{k_4 k_8 k_{11} k_{13}}(k_3)}{2 \cdot \dfrac{k_7 k_{12}}{k_4 k_8 k_{11}}(k_3)} \\ =& \frac{-\dfrac{k_4 k_8 k_{11}}{2 k_7 k_{12}}\left(1 + \dfrac{k_2 k_5 k_9 k_{12}(h_0 + p_0)}{k_6 k_{10} k_{11} k_{14}}\right) + \dfrac{k_1(g_0 + r_0)}{2 k_{13}(k_3)}}{k_3}. \end{aligned}$$

Thus, $s = \dfrac{-a + b k_3}{k_3} + \sqrt{\left(\dfrac{-a + b k_3}{k_3}\right)^2 + \dfrac{c}{k_3}} = -\dfrac{a}{k_3} + b + \dfrac{\sqrt{(-a + b k_3)^2 + c k_3}}{k_3}$ where

$$\begin{aligned} a =& \frac{k_4 k_8 k_{11}}{2 k_7 k_{12}}\left(1 + \frac{k_2 k_5 k_9 k_{12}(h_0 + p_0)}{k_6 k_{10} k_{11} k_{14}}\right), \\ b =& \frac{k_1(g_0 + r_0)}{2 k_{13}(k_3)} \text{ and,} \\ c =& \frac{k_1 k_4 k_8 k_{11}(g_0 + r_0)}{k_7 k_{12} k_{13}}. \end{aligned}$$

Then

$$\frac{ds}{dk_3} = \frac{a}{k_3^2} \cdot \frac{bk_3 - a - \dfrac{ck_3}{2a} + \sqrt{(-a+bk_3)^2 + ck_3}}{\sqrt{(-a+bk_3)^2 + ck_3}}.$$

Now, suppose the numerator of the second factor of $\dfrac{ds}{dk_3}$ $bk_3 - a - \dfrac{ck_3}{2a} + \sqrt{(-a+bk_3)^2 + ck_3} \leq 0$. Then,

$$bk_3 - a - \frac{ck_3}{2a} + \sqrt{(-a+bk_3)^2 + ck_3} \leq 0 \iff 4ab \leq c.$$

Substituting the values of a, b, and c, we obtain

$$4\left[\frac{k_4k_8k_{11}}{2k_7k_{12}}\left(1 + \frac{k_2k_5k_9k_{12}(h_0+p_0)}{k_6k_{10}k_{11}k_{14}}\right)\right]\left[\frac{k_1(g_0+r_0)}{2k_{13}(k_3)}\right] \leq \frac{k_1k_4k_8k_{11}(g_0+r_0)}{k_7k_{12}k_{13}},$$

which gives $1 + \dfrac{k_2k_5k_9k_{12}(h_0+p_0)}{k_6k_{10}k_{11}k_{14}} \leq 0$. This is impossible because all the values of the rate constants are positive and the initial concentrations are nonnegative. Therefore, $\dfrac{ds}{dk_3} > 0$ for all $k_3 > 0$ and hence, s is increasing over $k_3 > 0$. Similarly, we can show that s is increasing over k_7 and k_{11}, and decreasing over k_4, k_8, and k_{12}.

COMPILES: A computational package for deriving analytic positive steady states

Applying our method of getting the analytic positive steady states of a system via network decomposition and translation becomes challenging as the CRN becomes complex. The sheer number of reactions involved makes manual computation cumbersome. To resolve this, we developed a user-friendly, open-source, and publicly available computational package in MATLAB. Called COMPILES (COMPutIng anaLytic stEady States), the package automatically decomposes a CRN into its finest independent decomposition and combines the solutions of the subnetworks to get the analytic positive steady state solution of the whole system. It also gives additional information by enumerating the free parameters and conservation laws of the system.

Manual for the code

We now discuss how to enter the input and run the main code `steadyState.m` using a simple network. Details of the various functions used in the package can be found in README.txt in the GitHub repository.

Step 1) Make sure that the files `addReaction.m`, `edge.m`, `graph_.m`, and `vertex.m` are in the same working directory as `steadyState.m`.

Step 2) Create a structure named `model` by inputting a name for your model in `model.id`.

```
model.id = ‘Example 1’;
```

Step 3) Use the function `addReaction` to add the reactions of the CRN to the structure `model`. The string in the first line is just a visual guide to how the reaction looks. The second line lists the reactant species (strings), their respective stoichiometry (cell list), and their respective kinetic orders (array list). The third line is for the product species where we only fill out the third element (kinetic orders) if the reaction is reversible. Finally, the fourth line is either `true` or `false` depending on whether the reaction is reversible or not, respectively.

```
model = addReaction(model,‘X1+X2<->X3’, ...
                          {‘X1’, ‘X2’}, {1, 1}, [1], ...
                          {‘X3’}, {1}, [1], ...
                          true);
model = addReaction(model,‘X3->X3+X4’, ...
                          {‘X3’}, {1}, [1], ...
                          {‘X3’, ‘X4’}, {1, 1}, [ ], ...
                          false);
model = addReaction(model,‘X4->0’, ...
                          {‘X4’}, {1}, [1], ...
                          { }, { }, [ ], ...
                          false);
model = addReaction(model,‘X4+X5->X5’, ...
                          {‘X4’, ‘X5’}, {1, 1}, [1], ...
                          {‘X5’}, {1}, [ ], ...
                          false);
```

Step 4) Finally, simply run the function `steadyState` with the input `model` and outputs `equation`, `species`, `free_parameter`, `conservation_law`, and `model`. `equation` is a list consisting of the parametrization of the steady state of the system, `species` is a list of steady state species of the network, `free_parameter` is a list of free parameters of the steady state, `conservation_law` is a list of conservation laws of the system, and `model` is the structure of the complete network with all the species listed in the `species` field of the structure `model`.

```
[equation, species, free_parameter, conservation_law, model] = steadyState(model);
```

Output) When run, the function `steadyState` automatically outputs the number of subnetworks the finest independent decomposition has. In particular, the network has two independent subnetworks. Then, for each subnetwork, the reactions involved and the solution are displayed. For instance, Subnetwork 1 has two reactions $R_1 : X_1 + X_2 \to X_3$ and $R_2 : X_3 \to X_1 + X_2$. In this subnetwork, the steady states of species `X1` and `X3` are parametrized in terms of the rate constants and the free parameters `X2`, `X4`, and `X5`. Finally, the final solution using the combined solutions from each subnetwork is generated. Lists of free parameters and conservation laws are also displayed.

```
The network has 2 subnetworks.

- Subnetwork 1 -

R1:  X1+X2->X3
R2:  X3->X1+X2

Solving Subnetwork 1...

X1 = (k2*tau2)/(k1*tau1)
X2 = tau1
X3 = tau2

- Subnetwork 2 -

R3:  X3->X3+X4
R4:  X4->0
R5:  X4+X5->X5

Solving Subnetwork 2...
```

```
X3 = (tau3*(k4 + sigma1))/k3
X4 = tau3
X5 = sigma1/k5

Solving positive steady state parametrization of the entire network...

The solution is:

X1 = (X4*k2*(k4 + X5*k5))/(X2*k1*k3)
X3 = (X4*(k4 + X5*k5))/k3
Free parameters:  X2, X4, X5

Conservation laws:
X2 - X1 = X2,0 - X1,0
X1 + X3 = X1,0 + X3,0
X5 = X5,0
```

On the other hand, to demonstrate how COMPILES works for large networks, we consider the metabolic insulin signaling system, where the biochemical species are listed in Table B and reactions in Fig 4 in the main text.

```
The network has 10 subnetworks.

- Subnetwork 1 -

R1:  X2->X3
R2:  X3->X2
R3:  X5->X4
R4:  X4->X5
R5:  X3->X5
R6:  X5->X2
R7:  X2->X6
R8:  X6->X2
R9:  X4->X7
R10:  X7->X4
R11:  X5->X8
R12:  X8->X5
R15:  X7->X6
R16:  X8->X6

Solving Subnetwork 1...

X2 = (tau1*(k2 + k5)*(k4*k6*k10*k12 + k4*k6*k10*k16 + k4*k6*k12*k15 + k3*k9*k12*k15 + k4*k6*k15*k16
+ k4*k10*k11*k16 + k6*k9*k12*k15 + k3*k9*k15*k16 + k4*k11*k15*k16 + k6*k9*k15*k16 + k9*k11*k15*k16))/
(k1*k5*k11*(k4*k10 + k4*k15 + k9*k15))
X3 = (tau1*(k4*k6*k10*k12 + k4*k6*k10*k16 + k4*k6*k12*k15 + k3*k9*k12*k15 + k4*k6*k15*k16 +
k4*k10*k11*k16 + k6*k9*k12*k15 + k3*k9*k15*k16 + k4*k11*k15*k16 + k6*k9*k15*k16 + k9*k11*k15*k16))/
(k5*k11*(k4*k10 + k4*k15 + k9*k15))
X4 = (k3*tau1*(k10 + k15)*(k12 + k16))/(k11*(k4*k10 + k4*k15 + k9*k15))
X5 = (tau1*(k12 + k16))/k11
X6 = (tau1*(k2*k4*k6*k7*k10*k12 + k4*k5*k6*k7*k10*k12 + k1*k3*k5*k9*k12*k15 + k2*k4*k6*k7*k10*k16
+ k2*k4*k6*k7*k12*k15 + k1*k4*k5*k10*k11*k16 + k2*k3*k7*k9*k12*k15 + k4*k5*k6*k7*k10*k16 +
k1*k3*k5*k9*k15*k16 + k4*k5*k6*k7*k12*k15 + k2*k4*k6*k7*k15*k16 + k2*k4*k7*k10*k11*k16 +
k2*k6*k7*k9*k12*k15 + k3*k5*k7*k9*k12*k15 + k1*k4*k5*k11*k15*k16 + k2*k3*k7*k9*k15*k16 +
k4*k5*k6*k7*k15*k16 + k4*k5*k7*k10*k11*k16 + k5*k6*k7*k9*k12*k15 + k2*k4*k7*k11*k15*k16 +
k2*k6*k7*k9*k15*k16 + k3*k5*k7*k9*k15*k16 + k1*k5*k9*k11*k15*k16 + k4*k5*k7*k11*k15*k16 +
k5*k6*k7*k9*k15*k16 + k2*k7*k9*k11*k15*k16 + k5*k7*k9*k11*k15*k16))/(k1*k5*k8*k11*(k4*k10 +
k4*k15 + k9*k15))
X7 = (k3*k9*tau1*(k12 + k16))/(k11*(k4*k10 + k4*k15 + k9*k15))
X8 = tau1
```

```
- Subnetwork 2 -

R13:  0->X6
R14:  X6->0

Solving Subnetwork 2...

X6 = k13/k14

- Subnetwork 3 -

R17:  X9+X4->X10+X4
R18:  X9+X5->X10+X5
R19:  X10->X9

Solving Subnetwork 3...

X10 = (k18*tau2*tau3*(k17 + sigma1))/(k19*sigma1)
X4 = (k18*tau2)/sigma1
X5 = tau2
X9 = tau3

- Subnetwork 4 -

R20:  X10+X11->X12
R21:  X12->X10+X11

Solving Subnetwork 4...

X10 = (k21*tau5)/(k20*tau4)
X11 = tau4
X12 = tau5

- Subnetwork 5 -

R22:  X14+X12->X13+X12
R23:  X13->X14

Solving Subnetwork 5...

X12 = (k23*tau6)/(k22*tau7)
X13 = tau6
X14 = tau7

- Subnetwork 6 -

R24:  X15->X13
R25:  X13->X15

Solving Subnetwork 6...

X13 = (k24*tau8)/k25
X15 = tau8

- Subnetwork 7 -

R26:  X16+X13->X17+X13
R27:  X17->X16
```

```
Solving Subnetwork 7...

X13 = (k27*tau10)/(k26*tau9)
X16 = tau9
X17 = tau10

- Subnetwork 8 -

R28:  X18+X13->X19+X13
R29:  X19->X18

Solving Subnetwork 8...

X13 = (k29*tau12)/(k28*tau11)
X18 = tau11
X19 = tau12

- Subnetwork 9 -

R30:  X20->X21
R31:  X21->X20
R32:  X20+X17->X21+X17
R33:  X20+X19->X21+X19

Solving Subnetwork 9...

X17 = sigma2/k32
X19 = sigma3/k33
X20 = (k31*tau13)/(k30 + sigma2 + sigma3)
X21 = tau13

- Subnetwork 10 -

R34:  0->X20
R35:  X20->0

Solving Subnetwork 10...

X20 = k34/k35

Solving positive steady state parametrization of the entire network...

The solution is:

X2 = (k8*k13*(k2 + k5)*(k4*k6*k10*k12 + k4*k6*k10*k16 + k4*k6*k12*k15 + k3*k9*k12*k15 +
k4*k6*k15*k16 + k4*k10*k11*k16 + k6*k9*k12*k15 + k3*k9*k15*k16 + k4*k11*k15*k16 + k6*k9*k15*k16 +
k9*k11*k15*k16))/(k14*(k2*k4*k6*k7*k10*k12 + k4*k5*k6*k7*k10*k12 + k1*k3*k5*k9*k12*k15 +
k2*k4*k6*k7*k10*k16 + k2*k4*k6*k7*k12*k15 + k1*k4*k5*k10*k11*k16 + k2*k3*k7*k9*k12*k15 +
k4*k5*k6*k7*k10*k16 + k1*k3*k5*k9*k15*k16 + k4*k5*k6*k7*k12*k15 + k2*k4*k6*k7*k15*k16 +
k2*k4*k7*k10*k11*k16 + k2*k6*k7*k9*k12*k15 + k3*k5*k7*k9*k12*k15 + k1*k4*k5*k11*k15*k16 +
k2*k3*k7*k9*k15*k16 + k4*k5*k6*k7*k15*k16 + k4*k5*k7*k10*k11*k16 + k5*k6*k7*k9*k12*k15 +
k2*k4*k7*k11*k15*k16 + k2*k6*k7*k9*k15*k16 + k3*k5*k7*k9*k15*k16 + k1*k5*k9*k11*k15*k16 +
k4*k5*k7*k11*k15*k16 + k5*k6*k7*k9*k15*k16 + k2*k7*k9*k11*k15*k16 + k5*k7*k9*k11*k15*k16))
X3 = (k1*k8*k13*(k4*k6*k10*k12 + k4*k6*k10*k16 + k4*k6*k12*k15 + k3*k9*k12*k15 + k4*k6*k15*k16 +
k4*k10*k11*k16 + k6*k9*k12*k15 + k3*k9*k15*k16 + k4*k11*k15*k16 + k6*k9*k15*k16 + k9*k11*k15*k16))/
(k14*(k2*k4*k6*k7*k10*k12 + k4*k5*k6*k7*k10*k12 + k1*k3*k5*k9*k12*k15 + k2*k4*k6*k7*k10*k16 +
k2*k4*k6*k7*k12*k15 + k1*k4*k5*k10*k11*k16 + k2*k3*k7*k9*k12*k15 + k4*k5*k6*k7*k10*k16 +
k1*k3*k5*k9*k15*k16 + k4*k5*k6*k7*k12*k15 + k2*k4*k6*k7*k15*k16 + k2*k4*k7*k10*k11*k16 +
```

k2*k6*k7*k9*k12*k15 + k3*k5*k7*k9*k12*k15 + k1*k4*k5*k11*k15*k16 + k2*k3*k7*k9*k15*k16 +
k4*k5*k6*k7*k15*k16 + k4*k5*k7*k10*k11*k16 + k5*k6*k7*k9*k12*k15 + k2*k4*k7*k11*k15*k16 +
k2*k6*k7*k9*k15*k16 + k3*k5*k7*k9*k15*k16 + k1*k5*k9*k11*k15*k16 + k4*k5*k7*k11*k15*k16 +
k5*k6*k7*k9*k15*k16 + k2*k7*k9*k11*k15*k16 + k5*k7*k9*k11*k15*k16))
X4 = (k1*k3*k5*k8*k13*(k10 + k15)*(k12 + k16))/(k14*(k2*k4*k6*k7*k10*k12 + k4*k5*k6*k7*k10*k12 +
k1*k3*k5*k9*k12*k15 + k2*k4*k6*k7*k10*k16 + k2*k4*k6*k7*k12*k15 + k1*k4*k5*k10*k11*k16 +
k2*k3*k7*k9*k12*k15 + k4*k5*k6*k7*k10*k16 + k1*k3*k5*k9*k15*k16 + k4*k5*k6*k7*k12*k15 +
k2*k4*k6*k7*k15*k16 + k2*k4*k7*k10*k11*k16 + k2*k6*k7*k9*k12*k15 + k3*k5*k7*k9*k12*k15 +
k1*k4*k5*k11*k15*k16 + k2*k3*k7*k9*k15*k16 + k4*k5*k6*k7*k15*k16 + k4*k5*k7*k10*k11*k16 +
k5*k6*k7*k9*k12*k15 + k2*k4*k7*k11*k15*k16 + k2*k6*k7*k9*k15*k16 + k3*k5*k7*k9*k15*k16 +
k1*k5*k9*k11*k15*k16 + k4*k5*k7*k11*k15*k16 + k5*k6*k7*k9*k15*k16 + k2*k7*k9*k11*k15*k16 +
k5*k7*k9*k11*k15*k16))
X5 = (k1*k5*k8*k13*(k12 + k16)*(k4*k10 + k4*k15 + k9*k15))/(k14*(k2*k4*k6*k7*k10*k12 +
k4*k5*k6*k7*k10*k12 + k1*k3*k5*k9*k12*k15 + k2*k4*k6*k7*k10*k16 + k2*k4*k6*k7*k12*k15 +
k1*k4*k5*k10*k11*k16 + k2*k3*k7*k9*k12*k15 + k4*k5*k6*k7*k10*k16 + k1*k3*k5*k9*k15*k16 +
k4*k5*k6*k7*k12*k15 + k2*k4*k6*k7*k15*k16 + k2*k4*k7*k10*k11*k16 + k2*k6*k7*k9*k12*k15 +
k3*k5*k7*k9*k12*k15 + k1*k4*k5*k11*k15*k16 + k2*k3*k7*k9*k15*k16 + k4*k5*k6*k7*k15*k16 +
k4*k5*k7*k10*k11*k16 + k5*k6*k7*k9*k12*k15 + k2*k4*k7*k11*k15*k16 + k2*k6*k7*k9*k15*k16 +
k3*k5*k7*k9*k15*k16 + k1*k5*k9*k11*k15*k16 + k4*k5*k7*k11*k15*k16 + k5*k6*k7*k9*k15*k16 +
k2*k7*k9*k11*k15*k16 + k5*k7*k9*k11*k15*k16))
X6 = k13/k14
X7 = (k1*k3*k5*k8*k9*k13*(k12 + k16))/(k14*(k2*k4*k6*k7*k10*k12 + k4*k5*k6*k7*k10*k12 +
k1*k3*k5*k9*k12*k15 + k2*k4*k6*k7*k10*k16 + k2*k4*k6*k7*k12*k15 + k1*k4*k5*k10*k11*k16 +
k2*k3*k7*k9*k12*k15 + k4*k5*k6*k7*k10*k16 + k1*k3*k5*k9*k15*k16 + k4*k5*k6*k7*k12*k15 +
k2*k4*k6*k7*k15*k16 + k2*k4*k7*k10*k11*k16 + k2*k6*k7*k9*k12*k15 + k3*k5*k7*k9*k12*k15 +
k1*k4*k5*k11*k15*k16 + k2*k3*k7*k9*k15*k16 + k4*k5*k6*k7*k15*k16 + k4*k5*k7*k10*k11*k16 +
k5*k6*k7*k9*k12*k15 + k2*k4*k7*k11*k15*k16 + k2*k6*k7*k9*k15*k16 + k3*k5*k7*k9*k15*k16 +
k1*k5*k9*k11*k15*k16 + k4*k5*k7*k11*k15*k16 + k5*k6*k7*k9*k15*k16 + k2*k7*k9*k11*k15*k16 +
k5*k7*k9*k11*k15*k16))
X8 = (k1*k5*k8*k11*k13*(k4*k10 + k4*k15 + k9*k15))/(k14*(k2*k4*k6*k7*k10*k12 + k4*k5*k6*k7*k10*k12
+ k1*k3*k5*k9*k12*k15 + k2*k4*k6*k7*k10*k16 + k2*k4*k6*k7*k12*k15 + k1*k4*k5*k10*k11*k16 +
k2*k3*k7*k9*k12*k15 + k4*k5*k6*k7*k10*k16 + k1*k3*k5*k9*k15*k16 + k4*k5*k6*k7*k12*k15 +
k2*k4*k6*k7*k15*k16 + k2*k4*k7*k10*k11*k16 + k2*k6*k7*k9*k12*k15 + k3*k5*k7*k9*k12*k15 +
k1*k4*k5*k11*k15*k16 + k2*k3*k7*k9*k15*k16 + k4*k5*k6*k7*k15*k16 + k4*k5*k7*k10*k11*k16 +
k5*k6*k7*k9*k12*k15 + k2*k4*k7*k11*k15*k16 + k2*k6*k7*k9*k15*k16 + k3*k5*k7*k9*k15*k16 +
k1*k5*k9*k11*k15*k16 + k4*k5*k7*k11*k15*k16 + k5*k6*k7*k9*k15*k16 + k2*k7*k9*k11*k15*k16 +
k5*k7*k9*k11*k15*k16))
X9 = (X19*k14*k19*k21*k23*k29*(k2*k4*k6*k7*k10*k12 +
k4*k5*k6*k7*k10*k12 + k1*k3*k5*k9*k12*k15 + k2*k4*k6*k7*k10*k16 + k2*k4*k6*k7*k12*k15 +
k1*k4*k5*k10*k11*k16 + k2*k3*k7*k9*k12*k15 + k4*k5*k6*k7*k10*k16 + k1*k3*k5*k9*k15*k16 +
k4*k5*k6*k7*k12*k15 + k2*k4*k6*k7*k15*k16 + k2*k4*k7*k10*k11*k16 + k2*k6*k7*k9*k12*k15 +
k3*k5*k7*k9*k12*k15 + k1*k4*k5*k11*k15*k16 + k2*k3*k7*k9*k15*k16 + k4*k5*k6*k7*k15*k16 +
k4*k5*k7*k10*k11*k16 + k5*k6*k7*k9*k12*k15 + k2*k4*k7*k11*k15*k16 + k2*k6*k7*k9*k15*k16 +
k3*k5*k7*k9*k15*k16 + k1*k5*k9*k11*k15*k16 + k4*k5*k7*k11*k15*k16 + k5*k6*k7*k9*k15*k16 +
k2*k7*k9*k11*k15*k16 + k5*k7*k9*k11*k15*k16))/(X11*X14*X18*k1*k5*k8*k13*k20*k22*k28*(k12 + k16)*
(k3*k10*k17 + k4*k10*k18 + k3*k15*k17 + k4*k15*k18 + k9*k15*k18))
X10 = (X19*k21*k23*k29)/(X11*X14*X18*k20*k22*k28)
X12 = (X19*k23*k29)/(X14*X18*k22*k28)
X13 = (X19*k29)/(X18*k28)
X15 = (X19*k25*k29)/(X18*k24*k28)
X16 = (X17*X18*k27*k28)/(X19*k26*k29)
X20 = k34/k35
X21 = (k34*(k30 + X17*k32 + X19*k33))/(k31*k35)
Free parameters: X11, X14, X17, X18, X19

Conservation laws:
X11 + X12 = X,11 + X12,0
X13 + X14 + X15 = X13,0 + X14,0 + X15,0

```
X16 + X17 = X16,0 + X17,0
X18 + X19 = X18,0 + X19,0
X10 - X11 + X9 = X10,0 - X11,0 + X9,0
```

Underlying algorithm of the code

The package first uses the function `indepDecomp` based on the algorithm of [3] to generate the finest independent decomposition of the inputted network. The first part of the output displays how many subnetworks the decomposition has. The conservation laws are then computed by getting the kernel of the transpose of the stoichiometric matrix of the network. The code will display this at the end of the output.

To keep track of the progress of the code, a message is displayed saying which subnetwork is currently being solved. The reactions in the subnetwork are displayed before the subnetwork is solved using the function `analyticSolution`. If the subnetwork is already weakly reversible and has deficiency zero, the subnetwork is not translated. Otherwise, the function `GCRN` is used to get the translation of the subnetwork and, consequently, its generalized CRN. The function `findTranslations` based on [7] takes the first translation resulting in a weakly reversible network with kinetic deficiency of zero. If it takes too long (in our code, we set the time limit to be 10 seconds) to generate a translation, solving the subnetwork is skipped. Otherwise, the method in [8] is implemented to determine the solution of the subnetwork which is subsequently displayed.

The `tau`s solved in each subnetwork using the method in [8] represent free parameters in the subnetwork. To start combining the solutions of the subnetworks, these `tau`s are replaced by the species they represent so that the solutions of the subnetworks are in terms of free parameters. If possible, the `sigma`s, which represent the phantom edges, are also solved in terms of rate constants, steady state species, and free parameters.

At this point, we have a combined list of solutions for some of the species of the network. The total number of final solutions correspond to the number of steady state species of the entire network. The code keeps the solutions for those species that were solved only once. For each species that has multiple solutions coming from different subnetworks, these solutions are collected into a system of equations. The system is then solved.

Once the species are unique, COMPILES tries to make sure the right-hand side of each solution contains only rate constants and free parameters. If this is not possible because some steady state species still appear and some are dependent on each other, the unsimplified version is displayed and a message appears stating that at least two species are dependent on each other. This can usually be solved by renaming variables so that a different species is initially solved in some subnetwork. If everything goes well, the analytic steady state solution displayed contains only rate constants and free parameters. If a subnetwork was not solved, no consolidation of solutions can happen. A message is displayed stating that some subnetworks were not solved.

SUPPLEMENTARY TABLES

Species	Meaning
C	dCas9 complex
G	gene 1
H	gene 2
S	single guide RNA 1
T	single guide RNA 2
CS	dCas9 with single guide RNA 1 complex
CT	dCas9 with a single guide RNA 2 complex
P	dCas9 with a single guide RNA 1 complex (CS) specifically bound to gene H
R	dCas9 with single guide RNA 2 complex (CT) specifically bound to gene G

Table A. The species in the CRISPRi toggle switch model in Fig 3a left in the main text.

Species	Meaning
X_2	unbound surface insulin receptors
X_3	unphosphorylated once-bound surface receptors
X_4	phosphorylated twice-bound surface receptors
X_5	phosphorylated once-bound surface receptors
X_6	unbound unphosphorylated intracellular receptors
X_7	phosphorylated twice-bound intracellular receptors
X_8	phosphorylated once-bound intracellular receptors
X_9	unphosphorylated IRS-1
X_{10}	tyrosine-phosphorylated IRS-1
X_{11}	unactivated PI 3-kinase
X_{12}	tyrosine-phosphorylated IRS-1/activated PI 3-kinase complex
X_{13}	PI(3,4,5)P_3 out of the total lipid population
X_{14}	PI(4,5)P_2 out of the total lipid population
X_{15}	PI(3,4)P_2 out of the total lipid population
X_{16}	unactivated Akt
X_{17}	activated Akt
X_{18}	unactivated PKC-ζ
X_{19}	activated PKC-ζ
X_{20}	intracellular GLUT4
X_{21}	cell surface GLUT4

Table B. The species in the metabolic insulin signaling model in Fig 4a in the main text.

SUPPLEMENTARY FIGURES

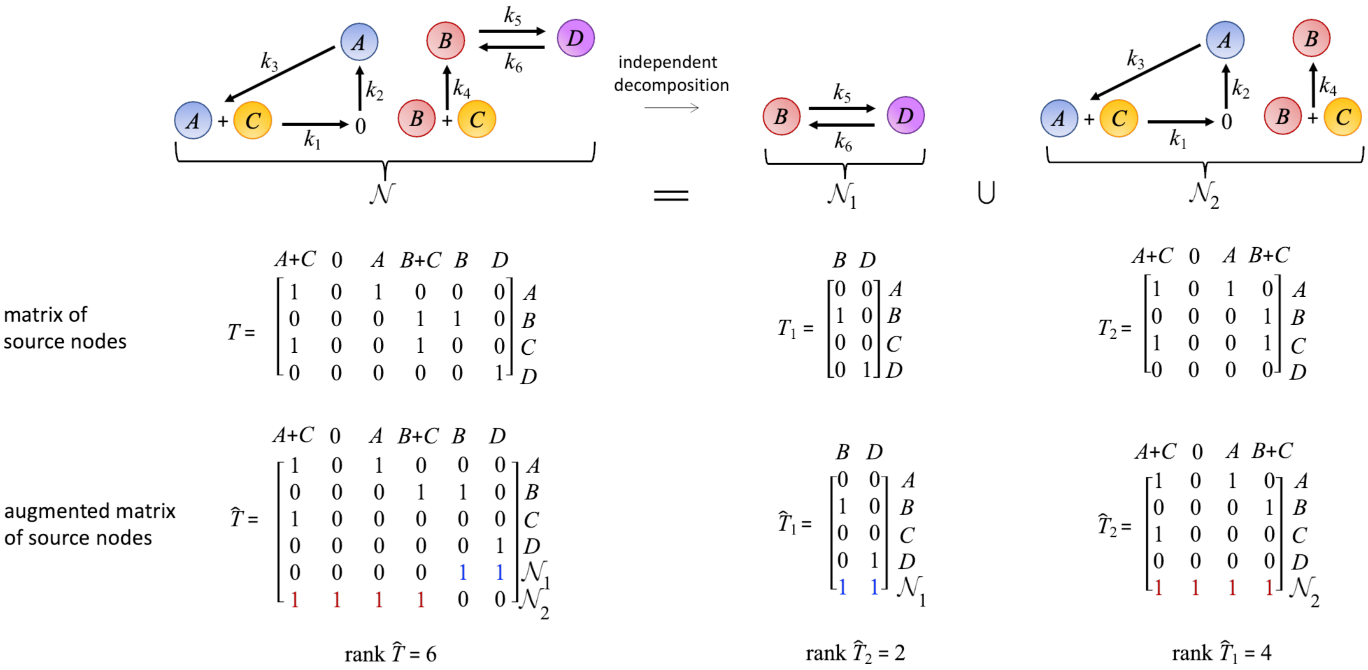

Fig A. **Computation of the augmented matrix of source nodes.** The CRN ($\mathcal{N}$) is decomposed into independent subnetworks ($\mathcal{N}_1$ and $\mathcal{N}_2$). Then, to get the matrix of source nodes of the whole network (T) and its subnetworks (T_1 and T_2), the stoichiometric coefficients of each species in each node are listed in each column. In particular, the first node in the whole network $\mathcal{N}$ is $A + C = 1A + 0B + 1C + 0D$. Hence, the stoichiometric coefficients 1, 0, 1, and 0 of species A, B, C, and D are listed in the first column of T. Then, to get the augmented matrix of source nodes of the whole network ($\widehat{T}$), the first step is to copy all the rows in the matrix of source nodes (T). The next step is to append w rows below this matrix where w is the number of subnetworks. Hence, we append two rows corresponding to the two subnetworks. Then, for each appended row associated with a subnetwork, we put 1 as the entry for those nodes that are present in the subnetwork. On the other hand, we put 0 for those that do not appear in this subnetwork. In particular, since B and D are nodes in the subnetwork associated with $\mathcal{N}_1$, then all the entries associated with these nodes are 1. On the other hand, nodes $A + C$, 0, A, and $B + C$ do not appear in $\mathcal{N}_1$, so the associated entries for both of these nodes are 0.

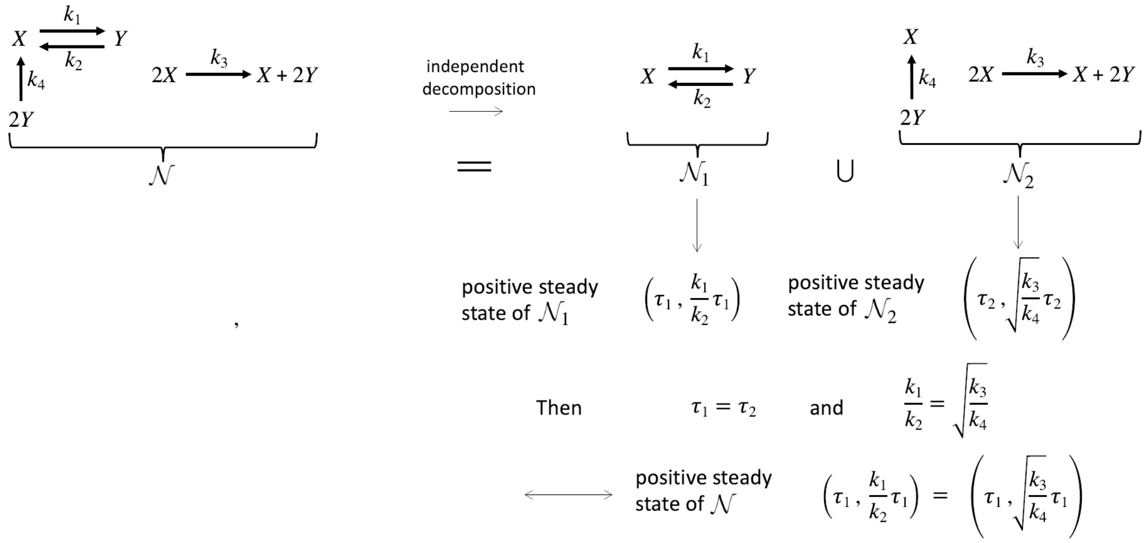

Fig B. **A CRN with a positive steady state for limited values of rate constants.** The CRN ($\mathcal{N}$) is decomposed into independent subnetworks ($\mathcal{N}_1$ and $\mathcal{N}_2$). Then, the positive steady states are computed for each subnetwork, i.e., $\left(\tau_1, \frac{k_1}{k_2}\tau_1\right)$ for $\mathcal{N}_1$ and $\left(\tau_2, \sqrt{\frac{k_3}{k_4}}\tau_2\right)$ for $\mathcal{N}_2$. Merging the positive steady states of these two subnetworks gives $\left(\tau_1, \frac{k_1}{k_2}\tau_1\right)$ or $\left(\tau_1, \sqrt{\frac{k_3}{k_4}}\tau_1\right)$ as the positive steady state of the CRN if $\frac{k_1}{k_2} = \sqrt{\frac{k_3}{k_4}}$. Otherwise, the CRN has no positive steady state. Hence, $\mathcal{N}$ has a positive steady state for limited rate constants only.

[1] M. Feinberg, Chemical reaction network structure and the stability of complex isothermal reactors I: The deficiency zero and deficiency one theorems, Chem. Eng. Sci. **42**, 2229 (1987).

[2] M. Feinberg, *Foundations of Chemical Reaction Network Theory* (Springer International Publishing, 2019).

[3] B. Hernandez and R. De la Cruz, Independent decompositions of chemical reaction networks, Bull. Math. Biol. **83**, 76 (2021).

[4] B. Hernandez, D. Amistas, R. De la Cruz, L. Fontanil, A. de los Reyes, and E. Mendoza, Independent, incidence independent and weakly reversible decompositions of chemical reaction networks, MATCH Commun. Math. Comput. Chem. **87**, 367 (2022).

[5] P. Lubenia, INDECS: INdependent DECComposition of networkS, Available online:, `https://github.com/pvnlubenia/INDECS`.

[6] B. Boros, On the positive steady states of deficiency one mass action systems, PhD thesis, Eötvös Loránd University (2013), `https://web.cs.elte.hu/~bboros/bboros_phd_thesis.pdf`.

[7] H. Hong, B. Hernandez, J. Kim, and J. K. Kim, Computational translation framework identifies biochemical reaction networks with special topologies and their long-term dynamics, SIAM J. Appl. Math. (2023).

[8] M. Johnston, S. Müller, and C. Pantea, A deficiency-based approach to parametrizing positive equilibria of biochemical reaction systems, Bull. Math. Biol. **81**, 1143 (2019).
