# Supplementary material for: A framework for deriving analytic steady states of biochemical reaction networks
Source: PLoS Comput Biol. 2023 Apr 13;19(4):e1011039. doi: 10.1371/journal.pcbi.1011039 (PMC10129002; doi:10.1371/journal.pcbi.1011039)
Supplement: Attachment — Submitted filename: Referee_report_Hernandez_et_al.pdf [file pcbi.1011039.s002.pdf]

Referee report

Hernandez et al

A framework for deriving analytic long-term behavior of biochemical reaction networks

PCOMPBIOL-D-22-01802

The authors develop a software package to symbolically parametrize the set of steady states of chemical reaction networks (CRNs) with mass-action kinetics.

Theoretically, their approach is based on three ingredients:

1. network decomposition (into independent subnetworks)
2. network translation (into dynamically equivalent generalized chemical reaction networks (GCRNs))
3. previous results on GCRNs

Having such a software package is definitely useful for the CRN community and modelers in systems biology. However, items 2 and 3 are not (yet) described clearly enough and impossible to understand (for readers without background in GCRNs – and even for me, as a developer of GCRN theory). This should be improved "everywhere": in abstract, intro, results, methods, ...

Another obvious observation concerns the title – it promises too much: Computing steady states is not the same as determining the "analytic long-term behavior". What about stability of steady states? (And periodic orbits? Homo/heteroclinic orbits? Chaos?) The title should be modified, and the stability issue should be discussed; see e.g. the recent paper Müller/Regensburger (2022) arxiv:2212.11039.

In my opinion, the paper can be published after a major revision.

Major issues (mostly related to GCRNs)

Abstract: line 22: ... into weakly reversible and deficiency zero generalized networks

Intro: line 76: ... into WR and DZ generalized networks

Intro: line 86: same issue

...

Please clarify "everywhere"!

Results: paragraphs 110–120 and 120–135: the description of item 2 (network translation into dynamically equivalent GCRNs) in Figs 1 and 2 is impossible to follow.

Please improve the presentation! (See my suggestions on the next page!)

Results: remaining paragraphs 135-210: the description of item 3 (main results for GCRNs) is missing in the Methods section. So the "description in words" (of effective deficiency, kinetic deficiency, "free parameters", tree constants, particular solution, generalized inverse, parametrization, ...) in the Results section is impossible to understand.

Please extend the Methods section, e.g. as in Johnston et al (2019) Bull Math Biol, state **all** results of GCRN theory that you are using, and refer to them in the Results section!

From my point of view, details of GCRNs and their theory are “hidden” in this manuscript (to make to presentation look simpler?), but in fact, those details would allow to follow the presentation more easily. To this end, the authors should introduce a bit more terminology, as follows:

Given a CRN (with graph G), a (dynamically equivalent) GCRN $(G', y, \tilde{y})$ is a graph G' and two maps y and $\tilde{y}$. It gives rise to two <u>associated</u> CRNs, namely a stoichiometric one (G', y) and a kinetic-order one $(G', \tilde{y})$. These three objects are constantly confused in the presentation, and should be referred to properly.

Given the network $\mathcal{N}$ used in Figs 1 and 2, I suggest to show the GCRN $\mathcal{N}'$ already in Fig 1, and Fig 2 can be used to demonstrate GCRN theory, via the <u>associated</u> CRNs $\mathcal{N}'\mathcal{S}$ and $\mathcal{N}'\mathcal{K}$. Of course, you can choose different (but unique) names and symbols for the crucial objects. For clarity, I made a sketch of the three networks that should appear in Figs 1 and 2 (and referred to in the main text of the Results section).

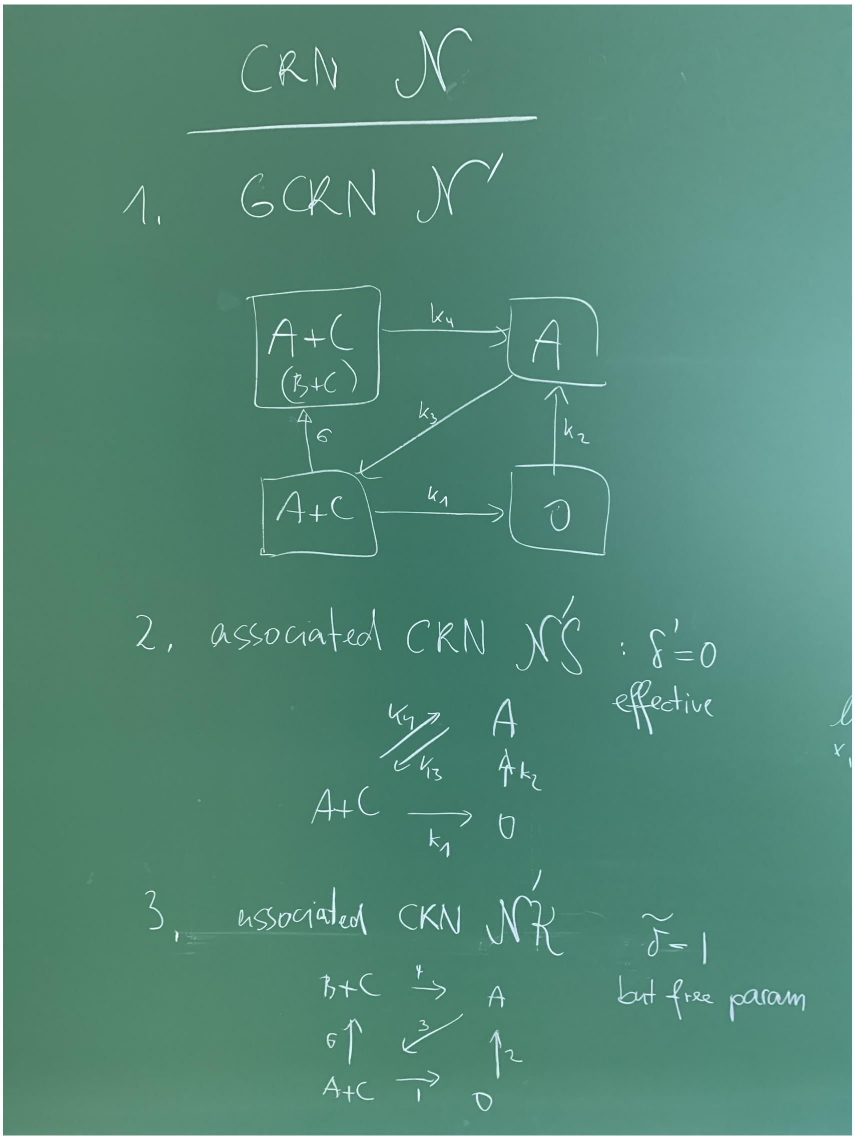

Also Fig 3 should be modified accordingly and described in proper theoretical terms.

As stated above, please include in the Methods section all parts of theory you are using: network decomposition, network translation into GCRNs, and GCRN theory.

Minor issues

Since my comments require considerable rewriting, I will list remaining typos (if any) after revision.
