# Supplementary material for: A framework for deriving analytic steady states of biochemical reaction networks
Source: PLoS Comput Biol. 2023 Apr 13;19(4):e1011039. doi: 10.1371/journal.pcbi.1011039 (PMC10129002; doi:10.1371/journal.pcbi.1011039)
Supplement: Attachment — Submitted filename: Response_letter.pdf [file pcbi.1011039.s003.pdf]

Response Letter

Response to Reviewer's Comments
We thank the reviewers for their constructive comments and helpful suggestions for strengthening the presentation and quality of our manuscript. Below, we give our detailed responses to the reviewers' comments. The reviewers' comments appear in **green**, and our responses in **black** while **blue** texts indicate the revised text of the manuscript.

Reviewer 1

Summary

The manuscript "A framework for deriving analytic long-term behavior of biochemical reaction networks" describes a mathematical approach for calculating the analytic steady states of a reaction network, realized as a system of (nonlinear) differential equations with polynomial right-hand side.

The manuscript is exceptionally well written, with many illuminating examples, clear explanations, and strong motivation. The mathematical approach is supported by the authors' new computational package, "COMPILES".

The manuscript makes significant progress in addressing an important and difficult mathematical/computational problem, and I strongly recommend that it be accepted for publication.

We appreciate the reviewer's kind comments.

Minor comment

In several places in the manuscript the general description of the importance of calculating the steady states analytically is based on the fact that "long-term behaviors of biochemical systems are described by their steady states". While this is of course true for many reaction networks, it may not be applicable for the cases where there are other attracting sites, such as oscillations, or boundary steady states, etc. I suggest the minor change that "long-term behaviors of biochemical systems are often described by their steady states" (I inserted the word "often", to suggest that there are some exceptions).

We revised accordingly by inserting the word "often" in the following lines of the manuscript:

Abstract (Line 18): The long-term behaviors of biochemical systems are often described by their steady states.

Author Summary (Line 39): Steady states often describe the long-term behaviors of biochemical systems, which are typically based on ordinary differential equations.

Introduction (Line 57): It is particularly important to determine properties of the steady states of ODEs because they often describe long-term behaviors of the CRNs.

Methods (Line 378): The long-term behavior of a system is often described by *steady states*, which could be solved by equating each time derivative to zero, i.e., $\frac{da}{dt} = \frac{db}{dt} = 0$, and solving for the concentrations of the species in terms of the rate constants.

Reviewer 2 (Dr. Pavel Kraikivski)

Summary

In the manuscript "A framework for deriving analytic long-term behavior of biochemical reaction networks", Hernandez et al. have a computational framework to analyze a molecular interaction network by breaking it into smaller independent subnetworks to derive steady states of the system. Authors have applied their computational tool to analyze toggle switch and insulin signaling models. It is very useful method and the computational tool which can be broadly applied. Thus, this work is well suitable for PLoS Computational Biology readers.

We appreciate the kind comments of the reviewer Dr. Pavel Kraikivski.

Minor comment 1

If there are limitations when the molecular interaction network cannot be analyzed with this approach or limits for the computational package applicability, I suggest to add such information to the discussion section.

We thank the reviewer for pointing this out. We indicated the limitations of the study in the Discussion section (Lines 342-347) as follows:

Our work focuses on the derivation of steady states and its usefulness in analyzing biochemical systems. It would be an interesting future work to extend our framework to analyze the other long-term behaviors of networks, such as stability of the steady states [1,4,8], boundary steady states [2,7], and oscillations [3]. In particular, for linear stability analysis of the complex-balanced equilibria of generalized networks, Boros, Müller and Regensburger [5,17] have recently proposed an interesting approach.

Minor comment 2

In line 101: "$B + C \to C$", should it be $B + C \to B$ as Figure 1 shows this reaction?

We thank the reviewer for bringing this to our attention. The comment is correct and we have revised this, i.e., "$B+C \to C$" was changed to "$B+C \to B$". This is a typographical error for particular line only and will not affect the rest of the manuscript.

The revision is given as follows:

Results (Line 110): the reaction $B + C \to B$ is not contained in a cycle.

Reviewer 3

Summary

The authors develop a software package to symbolically parametrize the set of steady states of chemical reaction networks (CRNs) with mass-action kinetics. Theoretically, their approach is based on three ingredients:

1. network decomposition (into independent subnetworks)
2. network translation (into dynamically equivalent generalized chemical reaction networks (GCRNs))
3. previous results on GCRNs.

Having such a software package is definitely useful for the CRN community and modelers in systems biology.

We thank the reviewer for acknowledging the importance of computational packages in the CRN community and to modelers in systems biology.

However, items 2 and 3 are not (yet) described clearly enough and impossible to understand (for readers without background in GCRNs – and even for me, as a developer of GCRN theory). This should be improved "everywhere": in abstract, intro, results, methods, ...

We appreciate the comments to improve the clarity of the manuscript. We have addressed these in detail (see below).

Another obvious observation concerns the title – it promises too much: Computing steady states is not the same as determining the "analytic long-term behavior". What about stability of steady states? (And periodic orbits? Homo/heteroclinic orbits? Chaos?) The title should be modified, and the stability issue should be discussed; see e.g. the recent paper Müller/Regensburger (2022) arxiv:2212.11039. In my opinion, the paper can be published after a major revision.

We provided the following paragraph in the Discussion section (Lines 337-342) to state the limitations of the study:

Our work focuses on the derivation of steady states and its usefulness in analyzing biochemical systems. It would be an interesting future work to extend our framework to analyze the other long term behaviors of networks, such as stability of the steady states [1,4,8], boundary steady states [2,7], and oscillations [3]. In particular, for linear stability analysis of the complex-balanced equilibria of generalized networks, Boros, Müller and Regensburger [5, 17] have recently proposed an interesting approach.

Additionally, we also changed the title to: "A framework for deriving analytic steady states of biochemical reaction networks".

Major comment 1

Abstract: line 22: ... into weakly reversible and deficiency zero generalized networks

We followed the reviewer's comment and revised in the following manner:

Abstract, Line 22: Specifically, biochemical reaction networks are transformed into weakly reversible and deficiency zero generalized networks, which allows the derivation of their analytic steady states.

Intro: line 76: ... into WR and DZ generalized networks

This part of the introduction was revised. Hence, the phrase does not appear anymore. The revision is given as follows:

Introduction, Lines 75-77: Hence, the method of *network translation*, that can modify the network structure while maintaining the dynamics, has been developed [11–14, 19].

Intro: line 86: same issue as the previous comment
Please clarify "everywhere"!

We followed the reviewer's comment and revised in the following manner:

Introduction, Line 96: We then translate the subnetworks into WR and DZ GCRNs by shifting reactions.

We checked the whole manuscript and we corrected the following parts:

Results, Line 246: Then, for subnetworks that are not WR and DZ (i.e., $\mathcal{N}_1$ and $\mathcal{N}_2$), the network translation was performed to obtain WR and DZ generalized networks.

Discussion, Line 313: Using our approach, we can derive the steady state of a CRN with the mass-action kinetics if each independent subnetwork is either a WR and DZ network, or can be transformed into a WR and DZ generalized network.

Moreover, to clarify generalized networks, we provided the following paragraph in the Introduction (Lines 75-87):

In general, however, CRNs arising in applications are rarely WR or DZ. Hence, the method of *network translation*, that can modify the network structure while maintaining the dynamics, has been developed [11–14, 19]. It involves shifting reactions, i.e., swapping reactions for ones with the same stoichiometric differences while keeping the original reaction rates. For instance, a reaction $A+B \to B$ could be shifted to $A \to 0$ while we keep the rate of the original reaction $k_1 ab$ to maintain the original dynamics. This network translation leads to *generalized chemical reaction network* (GCRN) with two associated structures. The first structure is the network's *stoichiometric CRN* with nodes that include the new stoichiometric nodes A and 0 from the shifted reaction, and the *kinetic-order CRN* with nodes that include the original source node $A+B$ from the original reaction. The deficiencies of the former and the latter network structures of the translated network are known

as the *effective* and *kinetic deficiencies*, respectively. If the stoichiometric network is WR and effective deficiency zero, and the kinetic order network is WR and kinetic deficiency zero, the translated GCRN is WR and DZ. In this case, a parametrization of the steady states can be computed easily [14].

Major comment 2

Results: paragraphs 110–120 and 120–135: the description of item 2 (network translation into dynamically equivalent GCRNs) in Figs 1 and 2 is impossible to follow.

Please improve the presentation! (See my suggestions on the next page!)

We did not follow exactly the presentation provided in literature [14] to simplify the description for a wide audience/readers of computational scientists (biologists) of PLOS Computational Biology. Nevertheless, we believe that accurate description is more important as the reviewer pointed out. Thus, we have now followed the terminologies of the GCRN theory as suggested by the reviewer. In particular, we edited both Figs 1 and 2, their captions, and the corresponding text to include the stoichiometric and kinetic-order CRNs arising from the network translation as a generalized network as follows:

Results, Lines 119-131: The first subnetwork ($\mathcal{N}_1$) (Fig. 1b left) is a WR and DZ network. On the other hand, the second subnetwork ($\mathcal{N}_2$) (Fig. 1b middle) is neither WR nor DZ. Thus, we have to translate $\mathcal{N}_2$ to make it WR and DZ. Specifically, $\mathcal{N}_2$ is not WR because the reaction $B + C \to B$ does not belong to a cycle. Thus, we replace it with $A + C \to A$, having the same stoichiometry to obtain the network, via the process of network translation, referred to as the *stoichiometric CRN* and denoted by $\mathcal{N}_2'^S$ (Fig. 1b middle) (see the Methods section and [11] for details on network translation). We keep the original reaction rate (k_2bc) for $A + C \to A$ so that the dynamics does not change. However, as the network structure changes, and in particular, as all the reactions now belong to a cycle, the stoichiometric CRN is a WR network. Furthermore, the deficiency of the stoichiometric CRN, called the effective deficiency, is zero, i.e., EDZ, because it has three nodes ($n = 3$), one connected component ($\ell = 1$), and a stoichiometric matrix of rank two ($s = 2$) so that $\delta = 3 - 1 - 2 = 0$.

Results, Lines 120-135 were revised as follows:

Results, Lines 132-143: Next, we construct the *kinetic-order CRN*, which is denoted by $\mathcal{N}_2'^K$. To do this, we first take all the edges of $\mathcal{N}_2'^S$. Then, we fill 0 in the tail of the edge associated with k_2 (Fig. 2a (iii)) because the source node of the edge associated with k_2 in the original network ($\mathcal{N}_2$) is 0 (Fig. 2a (i)). Similarly, we fill A in the tail of the edge associated with k_3 (Fig. 2a (iii)). Because the source nodes of the edges with k_1 and k_4 are $A + C$ and $B + C$ in the original network (Fig. 2a (i)), two source nodes ($A + C$ and $B + C$) are now placed in a single node (Fig. 2a (iii)). To separate the two source nodes, a phantom edge, which is an edge with a free parameter (σ_1), is introduced in such a way that the resulting network is still WR (Fig. 2a (iv)) (see the Methods section and [13] for

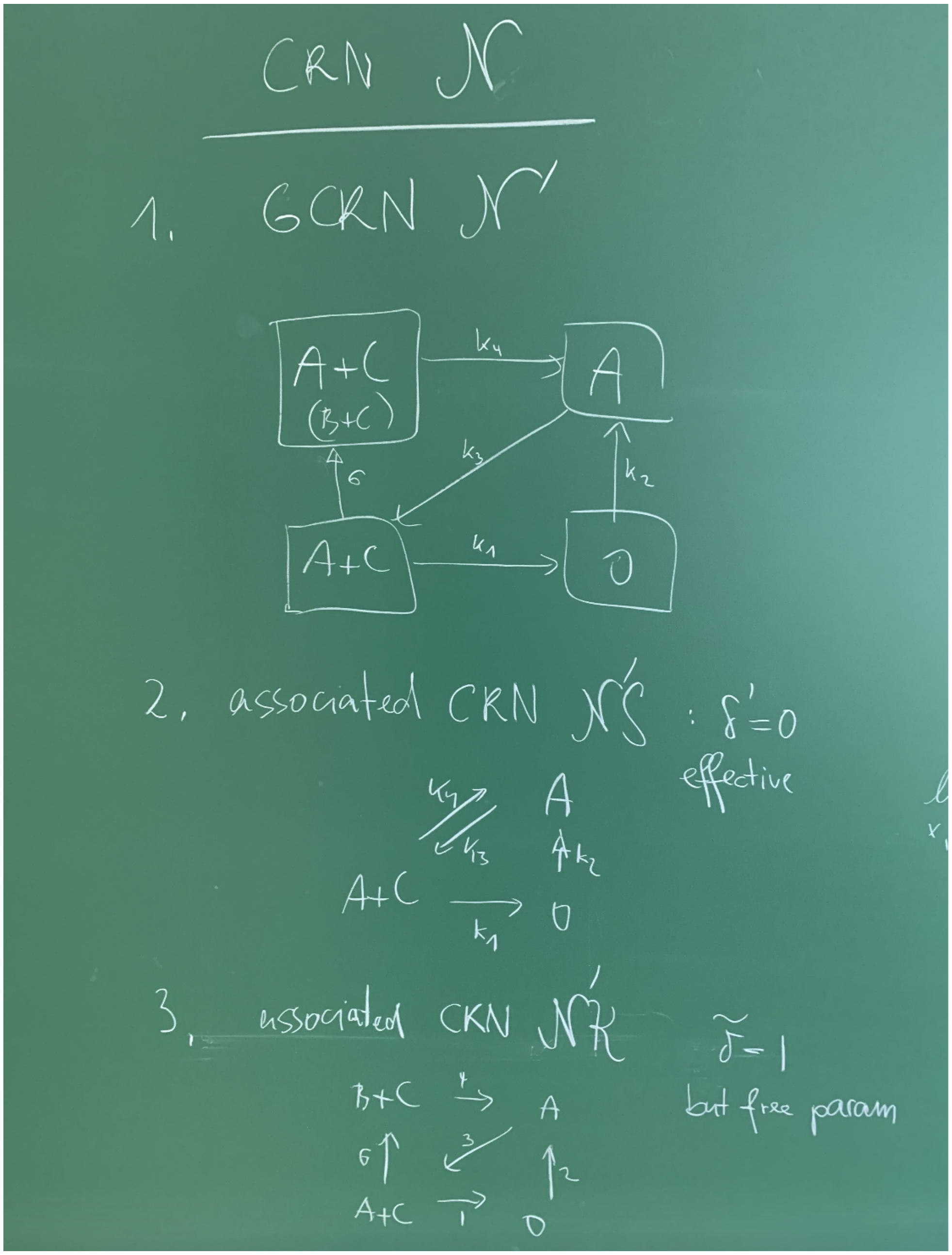

Figure 0: Figure made by Reviewer 3

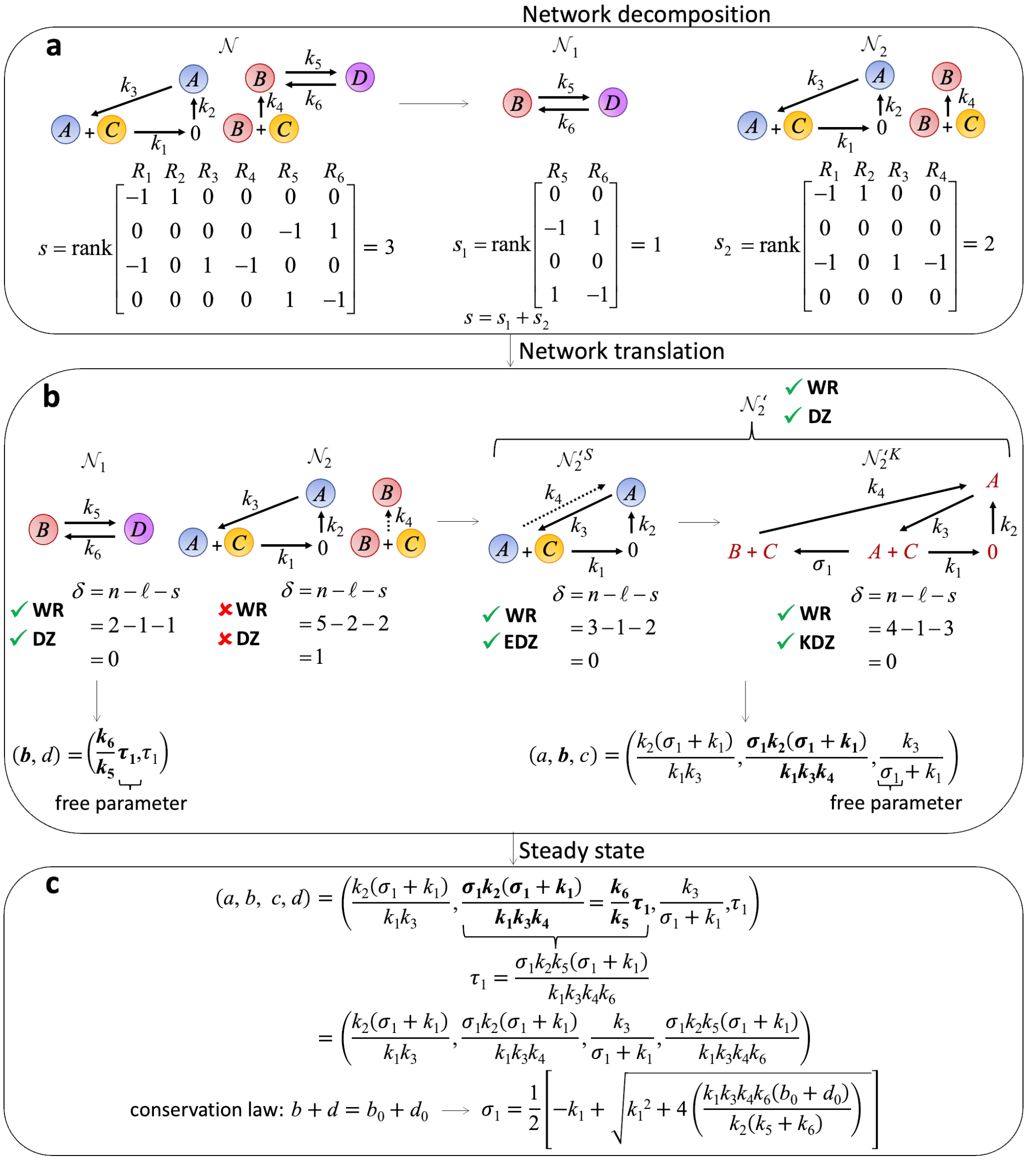

Figure 1: **Derivation of analytic steady state via network decomposition.** **a** The CRN ($\mathcal{N}$) is decomposed into independent subnetworks ($\mathcal{N}_1$ and $\mathcal{N}_2$) in such a way that the rank of the original matrix (s) is equal to the sum of the ranks of the stoichiometric matrices of subnetworks ($s_1 + s_2$). **b** Since the subnetwork $\mathcal{N}_2$ is not WR and DZ, network translation is performed. The translated subnework ($\mathcal{N}_2'$) is WR and DZ, i.e., its stoichiometric and kinetic-order CRNs ($\mathcal{N}_2'^S$ and $\mathcal{N}_2'^K$, respectively) are both WR and DZ, while its dynamics is equivalent to the dynamics of the original subnetwork ($\mathcal{N}_2$). Then, the steady states of the original subnetwork ($\mathcal{N}_1$) and the translated subnetwork ($\mathcal{N}_2'$) can be analytically derived since they are WR and DZ (see Fig. 2 for details). **c** The steady states of subnetworks are merged to identify the steady states of the original networks. In particular, the steady states of the common species of $\mathcal{N}_1$ and $\mathcal{N}_2$ (i.e., species B) are equated, which eliminates the free parameter τ_1. Then, by combining the steady state of every species, the steady state of the original network can be derived with one remaining free parameter σ_1. This σ_1 is computed from the conserved quantity in the network, which is the sum of the initial concentrations of species B and D (i.e., $b_0 + d_0$).

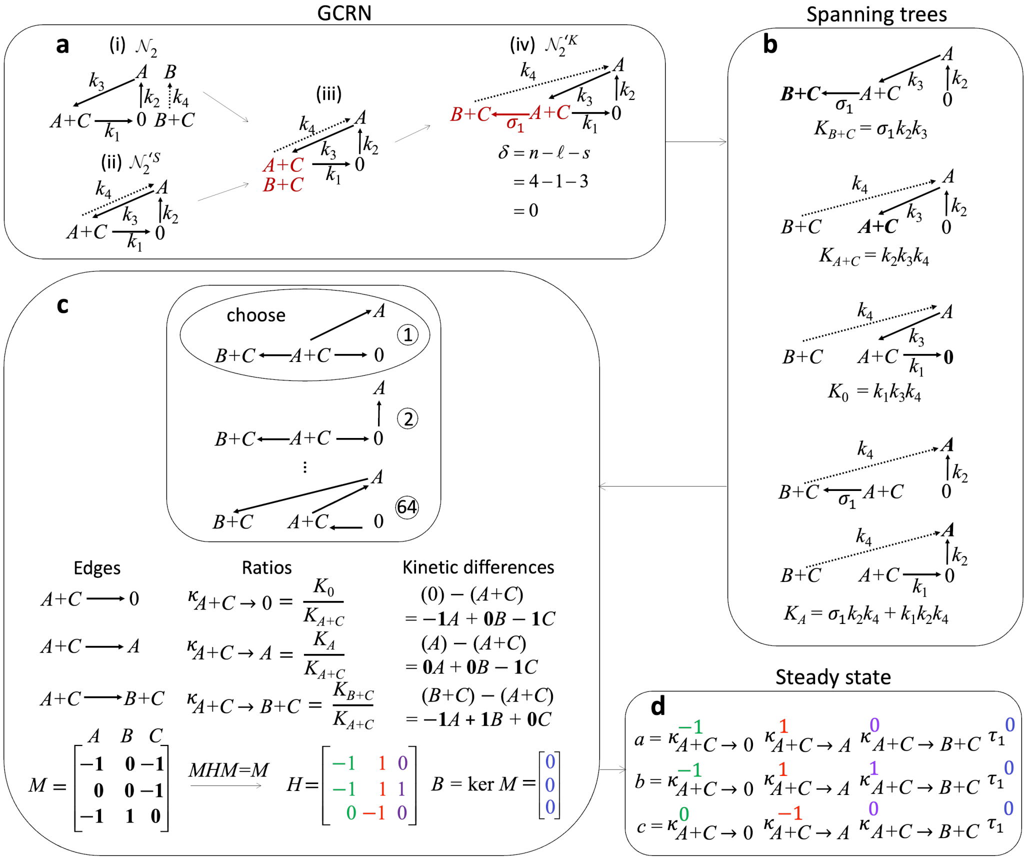

Figure 2: **Derivation of analytic steady state via network translation.** **a** The kinetic-order CRN $\mathcal{N}_2'^K$ is constructed from the edges of the stoichiometric CRN $\mathcal{N}_2'^S$ but the nodes are the source nodes of the associated reactions in the original network $\mathcal{N}_2$. For instance, the edges associated with k_1 and k_4 in $\mathcal{N}_2'^K$ (ii) have corresponding source nodes $A + C$ and $B + C$, respectively, in the original network (i). Since there are two source nodes ($A + C$ and $B + C$) which share a single node in (iii), a phantom edge with a zero stoichiometric vector and a free parameter rate constant (σ_1) is introduced (upper right). Notice that this operation maintains the dynamics of the original network. Proceed to the next step since the deficiency of $\mathcal{N}_2'^K$ is zero (i.e., KDZ). **b** Collect all the spanning trees of $\mathcal{N}_2'^K$ (i.e., connected subgraphs of the $\mathcal{N}_2'^K$ without a cycle) which direct towards each node ($B + C$, $A + C$, 0, and A). Then multiply the rate constants associated with the edges of each spanning tree. For instance, the products of the rate constants associated with the edges of the two spanning trees towards node A are $\sigma_1 k_2 k_4$ and $k_1 k_2 k_4$ (bottom). Compute each tree constant (K_i) by adding the product of the rate constants over all the spanning trees towards node (i). Hence, the tree constant $K_A = \sigma_1 k_2 k_4 + k_1 k_2 k_4$ is obtained. **c** Choose an arbitrary tree (i.e., a connected graph without a cycle) containing all the nodes in $\mathcal{N}_2'^K$, which is not necessarily its subgraph. Then, for each edge ($i \to i'$) of the chosen tree, find the ratios of the tree constants $\left(\kappa_{i\to i'} = \dfrac{K_{i'}}{K_i}\right)$ and the kinetic difference ($i' - i$) (middle). For instance, the edge $A + C \to 0$ has the ratio of the tree constants $\kappa_{A+C\to 0} = \dfrac{K_0}{K_{A+C}}$ and kinetic difference $(0) - (A + C) = -1A + 0B - 1C$. From the kinetic differences, construct the matrix M (bottom) by listing in each row the coefficients in the kinetic difference associated with an edge of the tree. Hence, the coefficients in the kinetic difference $-1A + 0B - 1C$ are the entries of the first row of M (i.e., $[-1, 0, -1]$). Then, compute a generalized inverse H of M (i.e., $MHM = M$) and the kernel B of M. **d** Derive the analytic steady state of the network from the ratio of the tree constants $\kappa_{i\to i'}$ and the matrices H and B. Specifically, raise the ratios of the tree constants $\kappa_{i\to i'}$ in a component-wise manner to the entries of a row of H, and get their product. For instance, the ratio of the tree constants $\kappa_{A+C\to 0}$, $\kappa_{A+C\to A}$, and $\kappa_{A+C\to B+C}$ are raised to the entries in the first row of H (-1, 1 and 0, respectively). This gives $\kappa_{A+C\to 0}^{-1}\kappa_{A+C\to A}^{1}\kappa_{A+C\to B+C}^{0}$. Meanwhile, one free parameter (τ_1) is introduced because the number of column of B is one. This τ_1 is raised to the entry of a row in B. Hence, τ_1^0 is obtained. Finally, get the product $\kappa_{A+C\to 0}^{-1}\kappa_{A+C\to A}^{1}\kappa_{A+C\to B+C}^{0}\tau_1^{0}$, which is the steady state value of species A. The steady state values for species B and C can be computed in a similar manner.

details on phantom edges). For the phantom edge, a zero stoichiometric vector is always assigned to maintain the dynamics of the original network. This produces a kinetic-order CRN $\mathcal{N}_2'^K$.

Major comment 3

Results: remaining paragraphs 135-210: the description of item 3 (main results for GCRNs) is missing in the Methods section. So the "description in words" (of effective deficiency, kinetic deficiency, "free parameters", tree constants, particular solution, generalized inverse, parametrization, ...) in the Results section is impossible to understand.

We added a section about the *generalized chemical reaction networks* in the Methods (Lines 408-445) section to discuss the Results section in a more understandable manner as suggested by the reviewer. This includes important descriptions, definitions and results that are essential in explaining the required concepts of effective and kinetic deficiencies, free parameters and previous results in parametrization of positive steady states of Johnston et al. [14]. In addition, we also described tree constant and generalized inverse in Theorem 2. Moreover, we indicated further references of tree constants in the Results section.

Generalized chemical reaction networks

In this section, we formally define the important concept of the generalized chemical reaction networks (GCRNs) pioneered by Müller and Regensburger [15, 16].

Let $G = (V, E)$ be a directed graph with vertex set V and edge set $E \subseteq V \times V$. Additionally, let $V_s = \{i | i \to j \in E\}$, which is the set of all the source vertices.

Definition 1 *A* generalized chemical reaction network *(GCRN) is a directed graph $G = (V, E)$ together with two maps*

1. *$y : V \to \mathbb{R}^m_{\geq 0}$ that assigns each vertex to a stoichiometric complex, and*
2. *$\widetilde{y} : V_s \to \mathbb{R}^m_{\geq 0}$ that assigns each source vertex to a kinetic complex.*

Given a CRN with an associated graph G, a dynamically equivalent GCRN (i.e., the associated ODEs of the GCRN agree with the ODEs of the original CRN) is a graph G' together with two maps y and $\tilde{y}$, and hence, with two sets of complexes. It gives rise to two associated CRNs, the stoichiometric one (G', y) and a kinetic-order one $(G', \widetilde{y})$. We call the deficiencies associated to the stoichiometric CRN and kinetic-order CRN, the *effective deficiency* and *kinetic deficiency*, respectively. If there is a map between the reactions of the original CRN and the stoichiometric CRN that preserves the reaction vectors and relates the source complexes in the original CRN to the kinetic complexes in the kinetic-order CRN, a GCRN is a *network translation* of a given CRN (see [11, 14]).

We now introduce the following definition of a phantom edge and then proceed with the important result of *parametrization of positive steady states* introduced by Johnston et al. (Theorem 2) (Theorems 15 and 14 of [14]) specifically in the sense of translated networks.

Definition 2 *For a given GCRN, we call an edge that connects identical stoichiometric complexes a* phantom edge. *Otherwise, we call it an* effective edge.

A phantom edge does not contribute to the associated ODEs because the associated stoichiometric complexes are identical. Hence, the associated dummy reaction rate constant, denoted by σ, can be arbitrary and can be considered a *free parameter*. We denote the sets of phantom edges and effective edges of the GCRN by E^0 and E^*, respectively.

Theorem 2 *Consider a weakly reversible translated network, which is a GCRN. Let $\mathcal{F}$ be any spanning forest containing all the nodes of the kinetic-order CRN. For each edge of $\mathcal{F}$, we define the kinetic difference as the vector produced by subtracting the head kinetic complex by the tail kinetic complex. Furthermore, M is the matrix containing all the kinetic differences as rows where the entries per row are arranged according to the order of the species. Let H be a generalized inverse of M (i.e., $MHM = M$). Finally, define B such that* $\text{im } B = \ker M$ *and* $\ker B = \{\mathbf{0}\}$. *Then, if the kinetic deficiency is zero, it follows that the set of parametrized complex balanced equilibrium is given by*

$$\bar{Z} = \left\{ \kappa(k^*, \sigma)^{H^\top} \circ \tau^{B^\top} | \sigma \in \mathbb{R}^{E^0}_{>0}, \, \tau \in \mathbb{R}^{m-\tilde{s}}_{>0} \right\} \neq \varnothing$$

where $\kappa(k^*, \sigma)^{H^\top} \circ \tau^{B^\top}$ is the Hadamard product (the number of components of κ is the total number of effective and phantom edges) with the component of κ associated with the edge $i \to i'$ as $\kappa_{i \to i'} = \dfrac{K_{i'}}{K_i}$ and tree constant *K_i as the sum (over all the spanning trees of the kinetic-order CRN towards node i) of the products of the rate constants associated with the edges of each spanning tree. In addition, if the effective deficiency is zero, then the set of positive steady states of the original network is precisely $\bar{Z}$.*

The theorem stated above covers the case when the kinetic deficiency is zero. For generalized networks with positive kinetic deficiency, additional conditions need to be checked before a steady state parametrization may be constructed (see Theorem 15 of [14]).

We also made revisions in paragraphs in lines 135-210 according to essential terms in the GCRN theory:

Results, Lines 144-222: Then, we compute the deficiency of $\mathcal{N}_2'^K$, i.e., the kinetic deficiency. The differences of the nodes of the edges except for the phantom edge are the same as the reaction vectors of the stoichiometric CRN, and the difference of the nodes of the phantom edge ($(B + C) - (A + C) = [-1, 1, 0, 0]^\top$) is independent from the reaction vectors of the stoichiometric CRN. Thus, the rank of the matrix of reaction vectors of $\mathcal{N}_2'^K$ is one more than the rank of the stoichiometric matrix of $\mathcal{N}_2'^S$ ($s = 3$). Furthermore, $\mathcal{N}_2'^K$ has one more node ($B + C$) compared to $\mathcal{N}_2'^S$ ($n = 4$). Additionally, the number of

connected components are the same ($\ell = 1$). Since $\delta = n - \ell - s = 4 - 1 - 3 = 0$, the kinetic deficiency of $\mathcal{N}_2'^K$ is zero (i.e., KDZ). We proceed to the next step as the kinetic deficiency is zero; otherwise, additional conditions need to be satisfied (see [13, 14]).

The stoichiometric CRN $\mathcal{N}_2'^S$ and kinetic-order CRN $\mathcal{N}_2'^K$ form the *translated network*, denoted by $\mathcal{N}_2'$, which is a generalized chemical reaction network (GCRN) (see the Methods section for details on GCRNs). $\mathcal{N}_2'$ is DZ because $\mathcal{N}_2'^S$ is EDZ and $\mathcal{N}_2'^K$ is KDZ. Furthermore, $\mathcal{N}_2'$ is WR because both $\mathcal{N}_2'^S$ and $\mathcal{N}_2'^K$ are WR. When $\mathcal{N}_2'$ is WR and DZ, the parametrization of the steady states can be easily calculated (see Theorem 2 in the Methods section).

To calculate the parametrization of the steady states, we need to get all the spanning trees of $\mathcal{N}_2'^K$, i.e., connected subgraphs without a cycle directed towards each node as shown in Fig. 2b. For instance, there is only one spanning tree directed towards $B + C$ because there is only one connected subgraph of the kinetic-order CRN without a cycle that points towards $B + C$ (Fig. 2a (iv)). Then, to get the tree constant (K_i) associated with a particular node i with only one spanning tree, we multiply the rate constants corresponding to the edges of this spanning tree (see the description of tree constants in Theorem 2 of the Methods section and [11] for more details). Hence, we obtain $K_{B+C} = \sigma_1 k_2 k_4$ as the tree constant directed towards the node $B + C$. In general, if there is more than one spanning tree directed towards a particular node, we have to repeat the process of getting the corresponding product for each spanning tree and then get the sum of the products over all the spanning trees directed towards the particular node. Specifically, there are two spanning trees directed towards node A because there are two connected subgraphs of $\mathcal{N}_2'^K$ without a cycle that point towards node A. Then, multiply the rate constants associated with the edges of each spanning tree. Hence, we obtain $\sigma_1 k_2 k_4$ and $k_1 k_2 k_4$ (bottom) as the products of the rate constants associated with the two spanning trees directed towards node A. Thus, the tree constant associated with node A is $K_A = \sigma_1 k_2 k_4 + k_1 k_2 k_4$. Note that the term "tree constant" was introduced in [11] to refer to the formulas following from the matrix tree theorem presented in [18] and adapted to chemical reaction network theory in [6].

Next, from $\mathcal{N}_2'^K$ (Fig. 2a (iv)), we form a tree (i.e., a connected graph without a cycle) that contains all the nodes of this CRN. The possible trees that can be formed are given in Fig. 2c (top). Then, for each edge of the tree, find the ratio of the tree constants (center) and the kinetic difference (middle right), the ratio of the tree constants $\kappa_{A+C\to 0} = \dfrac{K_0}{K_{A+C}}$, and an associated kinetic difference of $(0) - (A + C) = -1A + 0B - 1C$. After doing this for each edge of the tree, we construct the matrix M where each row corresponds to the vector of coefficients in the kinetic difference associated with each edge. For instance, the first row of M is the first kinetic difference $-1A + 0B - 1C = [-1, 0, -1]^\top$ associated with the first edge $A + C \to 0$. In addition, the second and third rows of M are precisely the vectors of coefficients in the kinetic differences associated with the two remaining edges of the tree. We then compute a generalized inverse H of M (i.e., $MHM = M$) and the kernel B of M.

From the matrices H and B together with the ratios of the tree constants, we can derive the

analytic steady state of the subnetwork $\mathcal{N}_2$ (Fig. 2d). In particular, to get the analytic steady state of the first species A, we raise each ratio of the tree constants associated with the three edges (i.e., $\kappa_{A+C\to 0}$, $\kappa_{A+C\to A}$, and $\kappa_{A+C\to B+C}$) to each of the entries in the first row of H (i.e., -1, 1, and 0, respectively), and then multiply them to obtain $\kappa^{-1}_{A+C\to 0} \cdot \kappa^{1}_{A+C\to A} \cdot \kappa^{0}_{A+C\to B+C}$. Additionally, we introduce a number of free parameters, as many as the number of columns of B. We have a single free parameter (τ_1) because there is only one column of B. We raise this free parameter to the value of the first row of B (i.e., 0) and we get τ_1^0. We multiply this value from the previously obtained product, which gives $(\kappa^{-1}_{A+C\to 0} \cdot \kappa^{1}_{A+C\to A} \cdot \kappa^{0}_{A+C\to B+C}) \cdot (\tau_1^0)$. This is the value of the analytic steady state of the first species A. By following the same procedure, we can get the analytic steady state values of the two remaining species of the translated subnetwork ($\mathcal{N}_2'$).

Because $\mathcal{N}_1$ is a WR and DZ network, we did not need the network translation. In this case, we can get its steady state by applying the procedure (Fig. 2) to $\mathcal{N}_1$. While we illustrate the parametrization of steady states with the simple example, a rigorous description is provided in the Methods section (see Theorem 2).

Then, we combine the steady states of the two subnetworks ($\mathcal{N}_1$ and $\mathcal{N}_2$) to derive the steady state of the original whole network $\mathcal{N}$. Specifically, we equate the steady state values of the common species of the two subnetworks. That is, since species B appears in both subnetworks, we equate the steady state values of species B in $\mathcal{N}_1$ $\left(\text{i.e., } \frac{k_6}{k_5}\tau_1\right)$ and $\mathcal{N}_2$ $\left(\text{i.e., } \frac{\sigma_1 k_2(\sigma_1 + k_1)}{k_1 k_3 k_4}\right)$ (Fig. 1b bottom). As a result, we get $\tau_1 = \frac{\sigma_1 k_2 k_5(\sigma_1 + k_1)}{k_1 k_3 k_4 k_6}$. By using this, we can eliminate the free parameter (τ_1) in the combined steady state (Fig. 1c upper left) and derive the analytic steady state of the whole network $\mathcal{N}$ (Fig. 1c) with one free parameter σ_1. This free parameter can be solved using the conservation law $b + d = b_0 + d_0$ where b_0 and d_0 are the initial concentrations of species B and D, respectively. That is, by substituting the steady state values of species B and D to the conservation law, we can solve for the value of σ_1 in terms of the conserved quantity $(b_0 + d_0)$. The closed form can be used to easily identify the critical features of a steady state, such as multistability and concentration robustness, which we will illustrate as follows.

Major comment 4

Please extend the Methods section, e.g. as in Johnston et al (2019) Bull Math Biol, state all results of GCRN theory that you are using, and refer to them in the Results section!

From my point of view, details of GCRNs and their theory are "hidden" in this manuscript (to make to presentation look simpler?), but in fact, those details would allow to follow the presentation more easily. To this end, the authors should introduce a bit more terminology, as follows:

Given a CRN (with graph G), a (dynamically equivalent) GCRN $(G', y, \tilde{y})$ is a graph G' and two maps y and $\tilde{y}$. It gives rise to two associated CRNs, namely a stoichio- metric one (G, y) and a kinetic-order one (G, y). These three objects are constantly confused in the presentation, and should be referred to properly.

Given the network $\mathcal{N}$ used in Figs 1 and 2, I suggest to show the GCRN $\mathcal{N}'$ already in Fig 1, and Fig 2 can be used to demonstrate GCRN theory, via the associated CRNs $\mathcal{N}'\mathcal{S}$ and $\mathcal{N}'\mathcal{K}$. Of course, you can choose different (but unique) names and symbols for the crucial objects. For clarity, I made a sketch of the three networks that should appear in Figs 1 and 2 (and referred to in the main text of the Results section).

Also Fig 3 should be modified accordingly and described in proper theoretical terms.

As stated above, please include in the Methods section all parts of theory you are using: network decomposition, network translation into GCRNs, and GCRN theory.

We thank the reviewer for these helpful and constructive comments.

We have extended the methods section to include all the results of the GCRN theory that we are using as we have responded to Major Comment 3.

Furthermore, we rephrased the paragraph suggested by the reviewer to introduce the stoichiometric and kinetic-order CRN structures of a GCRN to easily define the effective and kinetic deficiencies in Methods as follows:

Methods, Lines 417-422: Given a CRN with an associated graph G, a dynamically equivalent GCRN (i.e., the associated ODEs of the GCRN agree with the ODEs of the original CRN) is a graph G' together with two maps y and $\tilde{y}$, and hence, with two sets of complexes. It gives rise to two associated CRNs, the stoichiometric one (G', y) and a kinetic-order one $(G', \widetilde{y})$. We call the deficiency associated to the stoichiometric CRN and kinetic-order CRN, the *effective deficiency* and *kinetic deficiency*, respectively.

Figs 1 and 2 were revised accordingly to indicate the two structures of the given translated network. We indicated the stoichiometric and kinetic-order CRNs as described above.

Moreover, we modified Fig. 3 to include the stoichiometric and kinetic-order CRNs for the first two subnetworks that were translated. We also provided the proper terms such as effective and kinetic deficiencies in the caption. Furthermore, we clarified the GCRN terminologies in the text as follows:

Methods, Line 246: To do this, we decompose the model into seven independent subnetworks (Fig. 3a right). Then, for subnetworks that are not WR and DZ (i.e., $\mathcal{N}_1$ and $\mathcal{N}_2$), the network translation was performed to obtain WR and DZ generalized networks. The translated subnetworks $\mathcal{N}_1'$ and $\mathcal{N}_2'$ are WR and DZ because their stochiometric networks ($\mathcal{N}_1'^S$ and $\mathcal{N}_2'^S$, respectively) are WR and EDZ networks and kinetic order networks ($\mathcal{N}_1'^K$

and $\mathcal{N}_2'^K$, respectively) are WR and KDZ networks, while their dynamics are equivalent to the original subnetworks $\mathcal{N}_1$ and $\mathcal{N}_2$, respectively (Fig. 3b left).

The Methods section now includes the network translation into GCRNs, and important concepts that we used in the GCRN theory (see our response to Major Comment 3). Additionally, we specify the decomposition theorem introduced by Martin Feinberg in the Methods section.

Methods, Lines 396-407: It was shown in [9, 10] that when the underlying network decomposition of a reaction network is independent, then the set of positive steady states of the whole system is equal to the intersection of the sets of positive steady states of the subsystems as long as the same kinetics is followed by each reaction from the whole network down to its corresponding subnetwork. The theorem is given as follows (see the Supplementary Information for details).

Theorem 1 *Let $\mathcal{N}$ be a reaction network with kinetics $\mathcal{K}$ decomposed into subnetworks $\mathcal{N}_1, \mathcal{N}_2, \dots, \mathcal{N}_\alpha$ and $\mathcal{K}_i$ be the restriction of $\mathcal{K}$ to reactions in $\mathcal{N}_i$. Then*

$$E_1 \cap E_2 \cap \cdots \cap E_\alpha \subseteq E$$

where E is the set of positive steady states of the whole network while E_i is the set of positive steady states of subnetwork $\mathcal{N}_i$. Furthermore, if the network decomposition is independent, then equality holds, i.e.,

$$E_1 \cap E_2 \cap \cdots \cap E_\alpha = E.$$

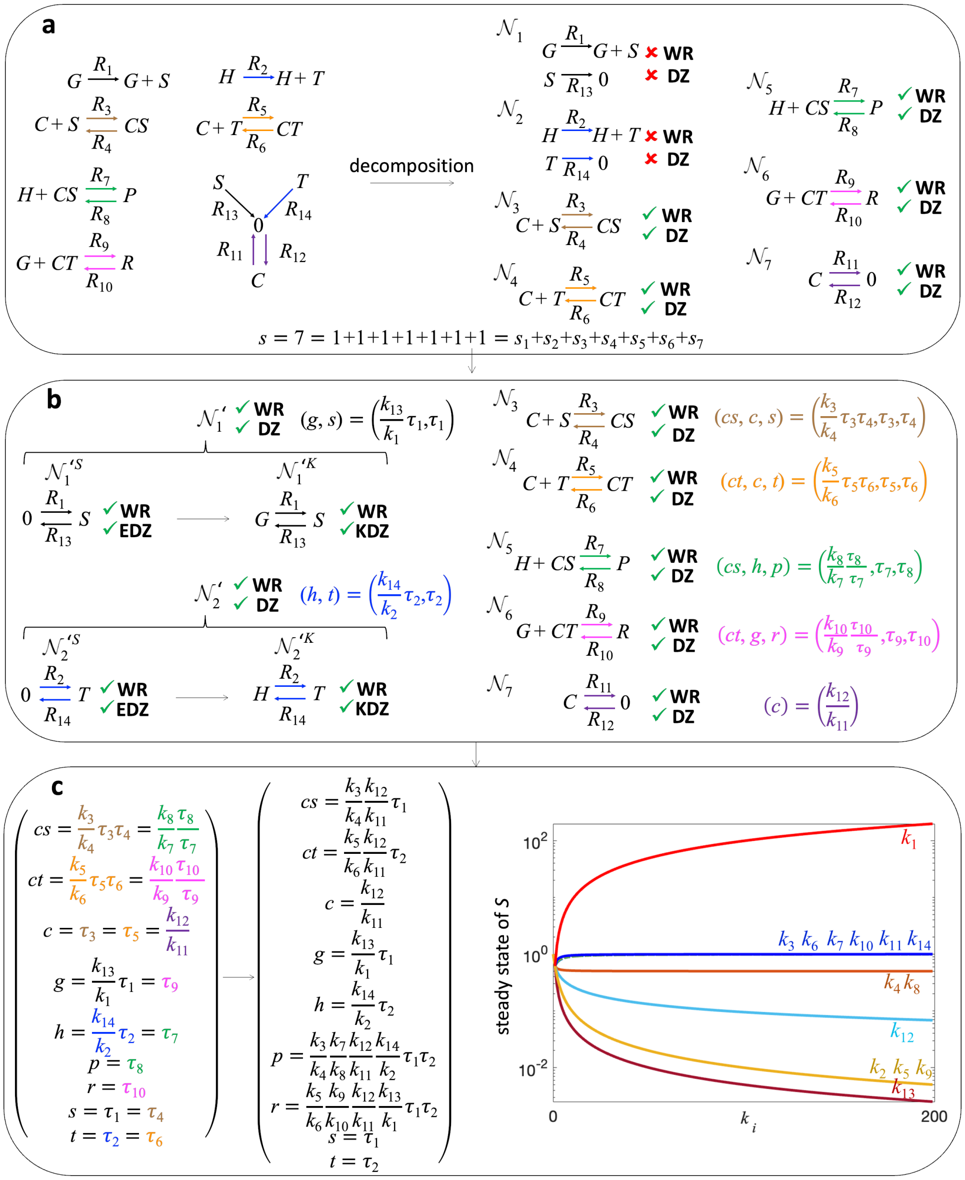

Figure 3: **Derivation of analytic steady state of a CRISPRi toggle switch model via network decomposition and network translation.** **a** A CRISPRi toggle switch network (left) that assumes dCas9 with a single guide RNA 1 (CS complex) and dCas9 with a single guide RNA 2 (CT complex) bind to a single site on their target genes H and G, respectively. The CRN has nine species (i.e., CS, CT, C, G, H, P, R, S, and T) and 14 reactions (i.e., R_1, ..., R_{14}), which is decomposed into seven independent subnetworks ($\mathcal{N}_1, \ldots, \mathcal{N}_7$) (right). **b** As the subnetworks $\mathcal{N}_1$ and $\mathcal{N}_2$ are not WR and DZ, network translation is performed. The translated subnetworks ($\mathcal{N}_1'$ and $\mathcal{N}_2'$) are WR and DZ (i.e., both its stoichiometric and kinetic-order CRNs are WR, and it is an EDZ and a KDZ generalized network) while their dynamics are equivalent to the dynamics of the original subnetworks ($\mathcal{N}_1$ and $\mathcal{N}_2$, respectively). Then, the steady states of the translated subnetworks ($\mathcal{N}_1'$ and $\mathcal{N}_2'$) and the original subnetworks ($\mathcal{N}_3, \ldots, \mathcal{N}_7$) can be analytically derived (see Fig. 2 for the outline of the steps). **c** The steady states of the subnetworks are combined by equating the steady states of the common species. For instance, the species CS is common to both subnetworks $\mathcal{N}_3$ and $\mathcal{N}_5$ so the steady state values of CS of both subnetworks are equated to each other (left). Then after solving the steady state of the whole network, two free parameters (τ_1 and τ_2) are left, which can be solved in terms of the conserved quantities. This analytic steady state solution could be used to determine the behavior of the steady state concentrations of species with respect to varying rate constants. In particular, the monotonicity of the steady state concentration of species S over varying rate constant k_i, when the rest of the rate constants are set to one, is illustrated on the right.

References

[1] M. Ali Al-Radhawi and D. Angeli. New approach to the stability of chemical reaction networks: piecewise linear in rates lyapunov functions. *IEEE Trans Automat Contr.*, 61:76–89, 2016.

[2] D. Anderson and D. Cappelletti. Discrepancies between extinction events and boundary equilibria in reaction networks. *J Math Biol.*, 79:1253–1277, 2019.

[3] M. Banaji. Inheritance of oscillation in chemical reaction networks. *Appl Math Comput.*, 325:191–209, 2018.

[4] B. Boros, J. Hofbauer, M. Banaji, S. Müller, and G. Regensburger. Planar S-systems: Global stability and the center problem. *Discrete Contin Dyn Syst Ser A*, 39:707–727, 2019.

[5] B. Boros, S. Müller, and G. Regensburger. Complex-balanced equilibria of generalized mass-action systems: necessary conditions for linear stability. *Math Biosci Eng.*, 17:442–459, 2020.

[6] G. Craciun, A. Dickenstein, A. Shiu, and B. Sturmfels. Toric dynamical systems. *J. Symbolic Comput.*, 44(11):1551–1565, 2009.

[7] G. Craciun, J. Helton, and R. Williams. Homotopy methods for counting reaction network equilibria. *Math Biosci.*, 216:140–149, 2008.

[8] P. Donnell and M. Banaji. Local and global stability of equilibria for a class of chemical reaction networks. *SIAM J Appl Dyn Syst.*, 12:899–920, 2013.

[9] M. Feinberg. Chemical reaction network structure and the stability of complex isothermal reactors I: The deficiency zero and deficiency one theorems. *Chem. Eng. Sci.*, 42:2229–2268, 1987.

[10] M. Feinberg. *Foundations of Chemical Reaction Network Theory.* Springer International Publishing, 2019.

[11] M. Johnston. Translated chemical reaction networks. *Bull. Math. Biol.*, 76:1081–1116, 2014.

[12] M. Johnston. A computational approach to steady state correspondence of regular and generalized mass action systems. *Bull. Math. Biol.*, 77:1065–1100, 2015.

[13] M. Johnston and E. Burton. Computing weakly reversible deficiency zero network translations using elementary flux modes. *Bull. Math. Biol.*, 81:1613–1644, 2019.

[14] M. Johnston, S. Müller, and C. Pantea. A deficiency-based approach to parametrizing positive equilibria of biochemical reaction systems. *Bull. Math. Biol.*, 81:1143–1172, 2019.

[15] S. Müller and G. Regensburger. Generalized mass action systems: Complex balancing equilibria and sign vectors of the stoichiometric and kinetic-order subspaces. *SIAM J. Appl. Math.*, 72:1926–1947, 2012.

[16] S. Müller and G. Regensburger. Generalized mass-action systems and positive solutions of polynomial equations with real and symbolic exponents (invited talk). In V. P. Gerdt, W. Koepf, W. M. Seiler, and E. V. Vorozhtsov, editors, *Computer Algebra in Scientific Computing. CASC 2014. Lecture Notes in Computer Science*, volume 8660, page 302–323. Springer, 2014.

[17] S. Müller and G. Regensburger. Sufficient conditions for linear stability of complex-balanced equilibria in generalized mass-action systems. arXiv, 2022.

[18] R. Stanley. *Enumerative Combinatorics, Volume 2.* Cambridge University Press, 1999.

[19] E. Tonello and M. Johnston. Network translation and steady-state properties of chemical reaction systems. *Bull. Math. Biol.*, 80:2306–2337, 2018.
