# Supplementary material for: A framework for deriving analytic steady states of biochemical reaction networks
Source: PLoS Comput Biol. 2023 Apr 13;19(4):e1011039. doi: 10.1371/journal.pcbi.1011039 (PMC10129002; doi:10.1371/journal.pcbi.1011039)
Supplement: Attachment — Submitted filename: Response_letter.pdf [file pcbi.1011039.s004.pdf]

# Response Letter

**Response to Reviewer's Comments**
We thank the reviewers for their constructive comments and helpful suggestions for strengthening the presentation and quality of our manuscript. Below, we give our detailed responses to the reviewer's comments. The reviewer's comments appear in **green**, and our responses in **black** while **blue** texts indicate the revised text of the manuscript.

# Reviewer 3

## Summary

The authors have addressed my major concerns and revised the manuscript accordingly. I recommend the manuscript for publication after fixing some remaining minor problems.

## Minor comment 1

line 184: please modify as follows: Then, for each edge of the tree, find the ratio of the tree constants (center) and the kinetic difference (middle right), **for example,** the ratio of ... , and **the** associated kinetic difference -1A+ ...

We revised accordingly as follows:

Lines 182-185: Then, for each edge of the tree, find the ratio of the tree constants (center) and the kinetic difference (middle right), for example, the ratio of the tree constants $\kappa_{A+C\to 0} = \dfrac{K_0}{K_{A+C}}$, and the associated kinetic difference of $(0)-(A+C) = -1A+0B-1C$.

## Minor comment 2

line 188: remove transpose (it is a row vector according to your convention)

The transposed row vector $[-1,0,-1]^\top$ was changed to $[-1,0,-1]$. We revised accordingly as follows:

Line 18: For instance, the first row of $M$ is the first kinetic difference $-1A+0B-1C = [-1,0,-1]$ associated with the first edge $A+C\to 0$.

## Minor comment 3

Fig 2 d: see my comments on $\tau_1^0$, below.
line 198: the kernel of M is trivial, that is, has dimension zero, so there is no free parameter $\tau_1$.

#free param = dim(ker(M))
The role of free monomial parameters is better explained in network $\mathcal{N}_1$.

Since there is no free parameter $\tau_1$ in the final answer, i.e., the steady state formula, we removed the sentence "We have a single free parameter ($\tau_1$) because there is only one column of $B$." Moreover, we clarified that indeed the steady state has no free parameter $\tau_1$ by mentioning that $\tau_1$ is raised to zero. Technically, we have an option not to mention a free parameter $\tau_1$ because and as the reviewer mentioned, the dimension of the kernel of the computed matrix $M$ is zero, i.e., all the entries are zero. Hence, in the end, this free parameter $\tau_1$ will just vanish because it will just be raised to zero. However, we chose to introduce $\tau_1$ to guide the reader on how the kernel of the matrix $M$ plays role in the computation of the steady-state formula especially for the case when some entries in the kernel of the matrix $M$ are nonzero.

Lines 199-203: We multiply this from the previously obtained product that gives $(\kappa^{-1}_{A+C\to 0} \cdot \kappa^{1}_{A+C\to A} \cdot \kappa^{0}_{A+C\to B+C}) \cdot (\tau_1^0) = \kappa^{-1}_{A+C\to 0} \cdot \kappa^{1}_{A+C\to A} \cdot \kappa^{0}_{A+C\to B+C}$, which is the value of the analytic steady state of the first species $A$ with no free parameter $\tau_1$ because $\tau_1$ is raised to zero.

## Minor comment 4

line 311: with mass-action kinetics

We revised accordingly as follows:

Line 311: Using our approach, we can derive the steady state of a CRN with mass-action kinetics if each independent subnetwork is either a WR and DZ network, or can be transformed into a WR and DZ generalized network.

## Minor comment 5

line 315: enormous numbers

We revised accordingly as follows:

Line 315: Previously, enormous numbers of numerical simulations were performed to investigate the bistability of the CRISPRi toggle switch model (Fig. 3) even within a limited range of parameters [26].

## Minor comment 6

line 333: an enormous amount

We revised accordingly as follows:

Line 333: Numerical approaches, however, typically involve an enormous amount of computation and investigate a limited range of parameter values.

## Minor comment 7

line 339: stability of steady states

We revised accordingly as follows:

Line 339: It would be an interesting future work to extend our framework to analyze the other long term behaviors of networks, such as stability of steady states [31−33], boundary steady states [34,35], and oscillations [36].

## Minor comment 8

line 342: Boros et al [] and Muller and Regensburger [] have recently proposed interesting approaches.

We revised accordingly as follows:

Lines 342-343: In particular, for linear stability analysis of the complex-balanced equilibria of generalized networks, Boros et al. [37] and Müller and Regensburger [38] have recently proposed interesting approaches.

## Minor comment 9

line 409: of generalized chemical reaction networks

We revised accordingly as follows:

Lines 410-411: In this section, we formally define the important concept of generalized chemical reaction networks (GCRNs) pioneered by Müller and Regensburger [12,44].

## Minor comment 10

line 415: that assigns to each vertex a stoichiometric complex

We revised accordingly as follows:

Line 416: that assigns to each vertex a stoichiometric complex

## Minor comment 11

line 416: that assigns to each vertex a kinetic complex

We revised accordingly as follows:

Line 417: that assigns to each vertex a kinetic complex.

## Minor comment 12

Theorem 2: the set of parametrized complex-balanced equilibria

We revised accordingly as follows:

Theorem 2 (below line 436): the set of parametrized complex-balanced equilibria

## Minor comment 13

line 436: (the number of components of $\kappa$ is the total number of effective and phantom edges) this is not correct. #components of $\kappa$ = number of edges in a spanning forest (which can be effective or phantom)

We revised accordingly as follows:

Lines 437-438: (the number of components of $\kappa$ is number of edges in a spanning forest, which can be effective or phantom)
